# Diffusion-Enhanced Förster Resonance Energy Transfer in Flexible Peptides: From the Haas-Steinberg Partial Differential Equation to a Closed Analytical Expression

**DOI:** 10.3390/polym15030705

**Published:** 2023-01-30

**Authors:** Maik H. Jacob, Roy N. D’Souza, Alexandra I. Lazar, Werner M. Nau

**Affiliations:** School of Science, Constructor University, 28759 Bremen, Germany

**Keywords:** fluorescence, FRET, peptide and polymer structure and dynamics, distance distribution, diffusion coefficient

## Abstract

In the huge field of polymer structure and dynamics, including intrinsically disordered peptides, protein folding, and enzyme activity, many questions remain that cannot be answered by methodology based on artificial intelligence, X-ray, or NMR spectroscopy but maybe by fluorescence spectroscopy. The theory of Förster resonance energy transfer (FRET) describes how an optically excited fluorophore transfers its excitation energy through space to an acceptor moiety—with a rate that depends on the distance between donor and acceptor. When the donor and acceptor moiety are conjugated to different sites of a flexible peptide chain or any other linear polymer, the pair could in principle report on chain structure and dynamics, on the site-to-site distance distribution, and on the diffusion coefficient of mutual site-to-site motion of the peptide chain. However, the dependence of FRET on distance distribution and diffusion is not defined by a closed analytical expression but by a partial differential equation (PDE), by the Haas-Steinberg equation (HSE), which can only be solved by time-consuming numerical methods. As a second complication, time-resolved FRET measurements have thus far been deemed necessary. As a third complication, the evaluation requires a computationally demanding but indispensable global analysis of an extended experimental data set. These requirements have made the method accessible to only a few experts. Here, we show how the Haas-Steinberg equation leads to a closed analytical expression (CAE), the Haas-Steinberg-Jacob equation (HSJE), which relates a diffusion-diagnosing parameter, the effective donor–acceptor distance, to the augmented diffusion coefficient, *J*, composed of the diffusion coefficient, *D*, and the photophysical parameters that characterize the used FRET method. The effective donor–acceptor distance is easily retrieved either through time-resolved or steady-state fluorescence measurements. Any global fit can now be performed in seconds and minimizes the sum-of-square difference between the experimental values of the effective distance and the values obtained from the HSJE. In summary, the HSJE can give a decisive advantage in applying the speed and sensitivity of FRET spectroscopy to standing questions of polymer structure and dynamics.

## 1. Introduction

There is no question about the significance of fluorescence in multiple molecular investigations; its speed and sensitivity even enable investigations on the level of the single molecule [1]. There is also no question about the significance of Förster resonance energy transfer in structural and dynamic molecular studies as the number of citations to Förster’s original paper approaches 10,000 [2]. We believe that diffusion-enhanced fluorescence resonance energy transfer, or DFRET, on the molecule ensemble can also develop into an influential method but is, as of now, difficult to handle and non-transparent in terms of data acquisition and analytics [3,4,5,6,7,8,9,10,11,12,13,14]. This article aims to make DFRET accessible to any scientist working in one of the many fields of polymer structure and dynamics. Here, we would like to point out that the Supporting Information file we have created contains assisting information in regard to different audiences: the layman, the expert, and, especially, the scientist who wants to put our method to work.

When a protein or peptide, or any molecule, has been modified at two sites with two fluorophoric moieties, one moiety can act as FRET donor and the other as FRET acceptor. When the intermittent chain between these sites is flexible, the optically excited donor and the acceptor can approach each other by diffusional motion. In accordance with Förster’s law (Equation (1)), the probability of energy transfer from donor to acceptor increases. Förster’s law connects the distance-dependent transfer rate constant, *k*_T_(*r*), with the rate constant of deactivation in the donor-only peptide, *k*_D_, with the Förster distance, *R*_0_, a constant that characterizes the donor-acceptor pair, and with the donor–acceptor distance, *R*_DA_.

(1) $$k_{T}=k_{D}{\left(\frac{R_{0}}{R_{\mathrm{DA}}}\right)}^{6}$$

**Figure 1:**
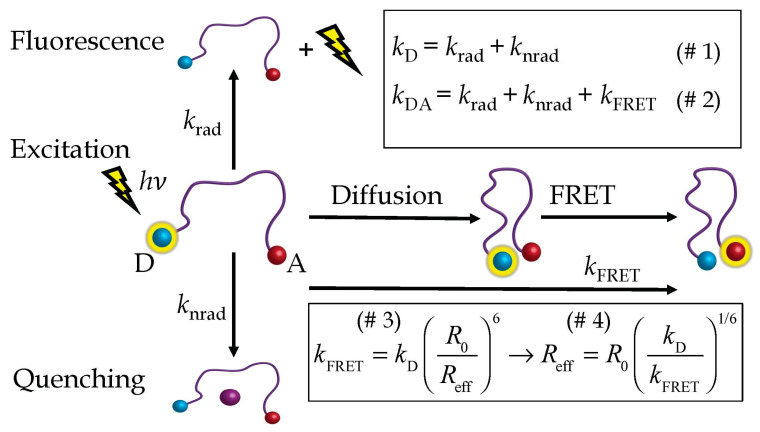
DFRET: Diffusion-enhanced Förster resonance energy transfer. After excitation, the excited donor, D, can either become deactivated by emitting fluorescence (*k*_rad_) or by being quenched, for instance, by iodine ions (*k*_nrad_) or by transferring its excitation energy to the acceptor, A (*k*_FRET_). FRET can take place at every D-A distance but is more likely to happen at shorter distances, which is why it is enhanced by D-A diffusion. The pertinent equations (I–IV) are explained in the main text.

Figure 1 gives a survey of the processes relevant in DFRET.

After excitation, the excited donor (D) can become deactivated in one of three ways: by emitting fluorescence with the rate constant *k*_rad_ (“*k*_radiative_”); by being quenched, for instance by iodine ions, with the rate constant *k*_nrad_ (“*k*_non-radiative_”); or by transferring its excitation energy to the acceptor (A) with the rate constant *k*_FRET_. In experimental measurements, we always use the donor-acceptor peptide (the DA-peptide) together with the “donor-only” peptide (the D-peptide). If no acceptor is present but only the donor, the measured rate of deactivation is *k*_D_ = *k*_rad_ + *k*_nrad_ (Figure 1; (I)). In the donor-acceptor peptide, in presence of an acceptor, the measured rate is *k*_DA_ = *k*_rad_ + *k*_nrad_ + *k*_FRET_ (Figure 1, (II)). Subtracting (I) from (II) yields the effective FRET rate, *k*_FRET_, *k*_FRET_ = *k*_DA_ − *k*_D_ (Equation (2)). In any normal situation, *k*_FRET_ increases with increasing diffusional motion between donor and acceptor. During the lifetime of the donor fluorescence, donor and acceptor can approach each other, and the probability of the FRET event increases in accordance with Förster’s law (Equation (1)), where *k*_T_ is the distance-dependent Förster transfer rate constant at a specific donor–acceptor distance, *R*_DA_. Consequently, *k*_FRET_ increases with diffusion, and the effective distance derived from *k*_FRET_ (Figure 1, (III), (IV), Equations (3) and (4)) decreases. In summary, the effective distance (Equation (4)) is a suitable diagnostic parameter to measure the diffusion enhancement of FRET. The effective distance serves as the interface between experiment and theory, that is, between the experimental results and the numerical solutions of the Haas-Steinberg equation (HSE). To restate, the effective distance is obtained experimentally from steady-state or time-resolved measurements on the peptide equipped with donor and acceptor, the “DA-peptide” (see Figure 1), and the peptide equipped only with the donor, the “D-peptide”. Measurements on the DA-peptide yield the fluorescence intensity, *I*_DA_, in steady-state experiments or the fluorescence decay rate, *k*_DA_, in time-resolved experiments, while measurements on the D-peptide yield *I*_D_ or *k*_D_. Exemplary chemical structures (Figure S1) and kinetics (Figure S2a,b) are given in the Supporting Information, Chapter 3.

(2) $$k_{\mathrm{FRET}}=k_{\mathrm{DA}}-k_{D}$$

(3) $$k_{\mathrm{FRET}}=k_{D}{\left(\frac{R_{0}}{R_{\mathrm{eff}}}\right)}^{6}$$

(4) $$R_{\mathrm{eff}}=k_{D}{\left(\frac{R_{0}}{k_{\mathrm{FRET}}}\right)}^{1/6}$$

### Introduction to the Haas-Steinberg Equation

The story of diffusion-enhanced FRET or DFRET started 40 years ago [3]. Since then, the number of published articles based on DFRET has remained small, not for lack of interest but for the method’s complexity [4,5,6,8,10,11,12,13,14]. In 1978, Steinberg derived what we began to call the Haas-Steinberg equation (HSE) [3]. Haas and co-workers developed unique techniques and used the HSE to tackle fundamental biological questions in protein folding, enzyme activity, and, more recently, in regard to intrinsically disordered peptides [3,5,10,11,15].

The HSE allows the calculation of theoretical effective distances. These theoretical or predicted values of HSE-based effective distances can then be fitted to the experimentally observed values in a multivariate analysis; in a global fit, that yields structure, i.e., the distance distribution, *p*(*r*), as well as dynamics, i.e., the diffusion coefficient, *D*.

In this work, we show how the HSE leads to a closed analytical equation, the HSJE (Haas-Steinberg-Jacob equation) by going through four equivalence statements (ES1-4). The HSJE eases and accelerates any global analysis, and, in addition, provides insight into the role of each parameter participating in DFRET.

## 2. Materials and Methods

The HSE was solved numerically by using the finite element method implemented in the PDE toolbox of MATLAB 2020 (MathWorks). The results from MATLAB calculations were analyzed within MATLAB 2020 and Microsoft Excel (Mac 2011). Figures were produced by using ProFit (QuantumSoft). For the global fits based on the HSJE and presented in the Supporting Information, Chapter 8, Figures S6 and S7, we used Microsoft Excel and its Solver add-in.

### 2.1. The HSE

The HSE can be written in various ways; we have specifically chosen the version shown in Equation (5).

(5) $$\frac{\partial N\left(r,t\right)}{\partial t}=-\frac{1}{\tau_{D}}N\left(r,t\right)-\frac{1}{\tau_{D}}\frac{R_{0}^{6}}{r^{6}}N\left(r,t\right)+\frac{\partial }{\partial r}\left(N_{0}\left(r\right)D\frac{\partial \left(N/N_{0}\right)}{\partial r}\right)$$

In Equation (5), we focus on the donor fluorescence lifetime, *τ*_D_, instead of the donor deactivation rate constant, *k*_D_, used in Figure 1 (Equation (I): *k*_D_ = *k*_rad_ + *k*_nrad_). It is much easier to recall the value of a lifetime, for instance, 100 ns, than that of a rate constant, for instance, 0.01 ns^−1^. The donor lifetime in the donor-only peptide, *τ*_D_, is simply the inverse of the rate constant, *k*_D_, in this peptide (Equation (6)).

(6) $$\tau_{D}=k_{D}^{-1}$$

Our shorthand notation for the HSE will simply be “(0) = (1) + (2) + (3)”, which enables us to address the left-hand-side term of the equation with term-(0) and the right-hand-side terms of the equation with terms-(1), -(2), and -(3). The function, *N*(*r*,*t*), on the left-hand side is a measure of the number of chains with excited donor after donor excitation. At a specific donor–acceptor distance, *r*, this number decreases with time, *t*. Thus, we have two independent variables, *r* and *t*, and, therefore, a partial differential equation, a PDE. *N*(*r*,*t*) is further normalized in a way that *N*_0_ = *N*(*r*, *t* = 0) in term-(3) is the initial probability density distance distribution. Upon irradiation, donors become activated with a probability assumed to be random, to be independent of the presence of an acceptor at various distances. In consequence, *N*_0_ is identical to the equilibrium distance distribution, *p*(*r*), of all chains present in the measurement sample. This distance distribution, *p*(*r*), as well as the site-to-site diffusion coefficient, *D*, in term-(3), is what we are interested in. They inform us on the structure and the dynamics of the (bio)polymer under investigation.

In summary, the HSE is a linear partial differential equation of second order: The second derivative becomes better visible on expansion of term-(3). Further, the HSE has constant coefficients that include the parameters, *D*, *τ*_D_, and *R*_0_. The HSE relates the rate of donor deactivation, term-(0); to the rate of donor deactivation in the absence of FRET, term-(1); plus the additional rate due to FRET, term-(2); plus the additional rate due to diffusion enhanced FRET, term-(3). A numerically obtained solution of the HSE yields the value of the effective distance between donor and acceptor. We elaborate on this calculation in Supporting Information, Chapter 4, Figure S3a–i.

### 2.2. Steinberg’s Derivation

Steinberg’s derivation of the HSE led him first to the following form (Equation (7)) [3].

(7) $$\frac{\partial N\left(r,t\right)}{\partial t}=-k_{D}N\left(r,t\right)-k_{D}\frac{R_{0}^{6}}{r^{6}}N\left(r,t\right)+\frac{\partial }{\partial r}\left(N_{0}\left(r\right)D\frac{\partial \left(N/N_{0}\right)}{\partial r}\right)$$

Here, the diffusion coefficient, *D*, in term-(3) does not depend on time, *t*, but it could still depend on the donor–acceptor distance, *r*: *D* = *f*(*r*). It is, however, even today, challenging to gather sufficient experimental results to investigate this possible dependence. Steinberg was content with an average diffusion coefficient that can be regarded as a constant. Then, *D* can be written in front of term-(3) (Equation (8)).

(8) $$\frac{\partial N\left(r,t\right)}{\partial t}=-k_{D}N\left(r,t\right)-k_{D}{\left(\frac{R_{0}}{r}\right)}^{6}N\left(r,t\right)+D\frac{\partial }{\partial r}\left(N_{0}\left(r\right)\frac{\partial \left(N/N_{0}\right)}{\partial r}\right)$$

When we apply Equation (6), we again obtain the HSE in the form of Equation (5). In summary, we know how to obtain *R*_eff_ from experiments as well as from numerically solving the HSE. We can therefore use the HSE to globally fit the experimental data. This was quite arduous in the past, where the HSE served a bit as a black box [5,6,10,11,12,13,14]. In the Results Section, we develop the Haas-Steinberg–Jacob equation (HSJE), a closed analytical expression (CAE). Any global fit based not on the HSE (PDE) but on the HSJE (CAE) is faster by about two orders of magnitude.

## 3. Results

### 3.1. Ideas and Concepts

**Figure 2:**
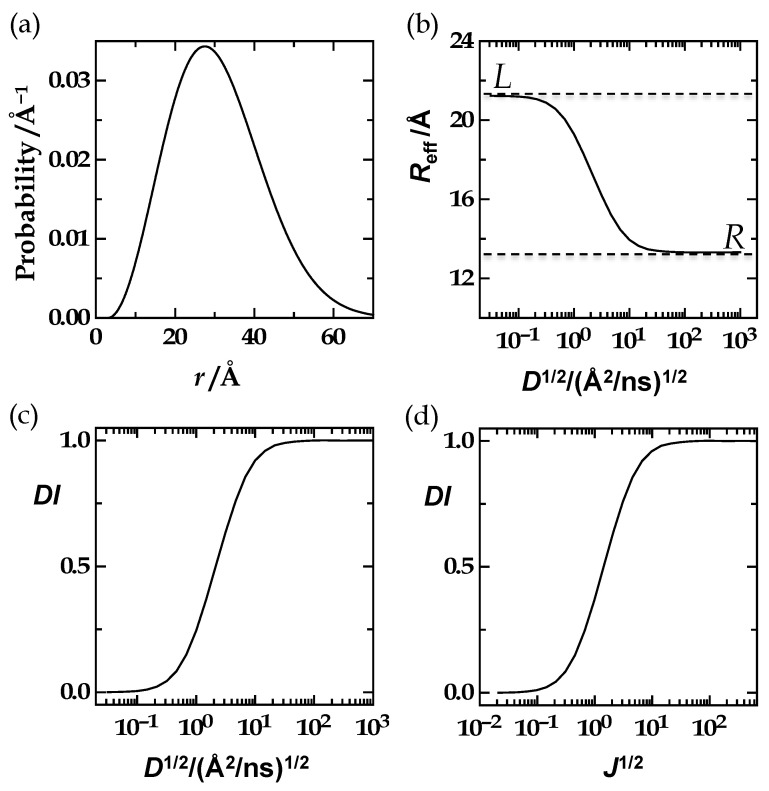
(**a**) An exemplary probability distance distribution of the donor–acceptor distance in a linear (bio)polymer (**b**) The diffusion profile or *R*_eff_ (*D*^1/2^) profile: Solving the HSE under variation of the diffusion coefficient results in values of the effective distance that range from *L* to *R* (dashed lines). The effective distance plotted against *D*^1/2^ approaches *R*_eff_ = *L* when the extent of diffusional motion approaches zero and approaches *R*_eff_ = *R* when the extent of diffusional motion approaches infinity. (**c**) The diffusion-influence profile or *DI*(*D*^1/2^) profile is obtained when the diffusion profile shown in (**b**) is normalized with *L* and *R* according to *DI* = (*R*_eff_ − *L*)/(*R* − *L*) (Equation (11)). The diffusion influence, *DI*, can adapt values between 0 (0%) and 1 (100%). (**d**) The diffusion-influence profile, the *DI*(*J*^1/2^) or *DI*(*X*) profile: The diffusion influence plotted against the square root of the augmented diffusion coefficient, *X* = *J*^1/2^ (see Equation (12)).

We first outline the basic ideas and concepts. Based on a freely selected distance distribution (Figure 2a) and selected values for the donor lifetime and Förster distance, *τ*_D_ and *R*_0_, we solved the HSE for a large range of values of the diffusion coefficient reaching from 10^−3^ to 10^6^ Å^2^/ns. The obtained effective-distance values formed a full diffusion profile, ranging from the “static limit” in the near absence of diffusion to the “dynamic limit” at high diffusion. In figures and graphs, we always plotted the effective distance against the square root of the diffusion coefficient or, later, against the square root of the “augmented diffusion coefficient” to keep the *x*-axis numbers or labels manageably small and readable (Figure 2b).

We have analytical equations for both extremes of the diffusion influence (Equations (9) and (10)): The “left” value, *L*, of the effective distance marks the static limit or the case of no diffusion (Equation (9)), as we first derived in the Supporting Information of reference [12]. The “right” value, *R*, of the effective distance marks the dynamic limit or the case of infinite diffusion (Equation (10)) [12,16] The derivations of the *L*- and *R*-equations can be found in this article’s Supporting Information, Chapters 4 and 4a,b. Explanations of the two underlying basic concepts, “the Energy Transfer Efficiency” and “the Average”, can be found in in the Supporting Information, Chapters 3 and 3a,b. At first glance, these equations appear to be quite complex, but, because of their analytical character, *L* and *R* are calculated within Matlab or Excel in a fraction of a second. We note that *L* is a function of the Förster radius and the distance distribution, while *R* is a function of only the distance distribution. Further, we note that any integration of *p*(*r*) takes place from the left-integration limit, *r*_L_, to the right-integration limit, *r*_R_. A physical but oversimplified perspective is to view *r*_L_ as the closest possible distance between donor and acceptor.

(9) $$L=R_{\mathrm{eff},D\to 0}=R_{0}{\left({\left(1-\int_{r_{L}}^{r_{R}}p(r)\left(\frac{r^{6}}{r^{6}+R_{0}^{6}}\right)dr\right)}^{-1}-1\right)}^{1/6}$$

(10) $$R=R_{\mathrm{eff},D\to \infty }={\langle r^{-6}\rangle }^{-1/6}={\left(\int_{r_{L}}^{r_{R}}\left(1/r^{6}\right)p(r)dr\right)}^{-1/6}$$

As we have these analytical equations for the static and dynamic limit of the effective distance, the *L* and *R* values are supposed to be extremely close to the effective-distance values at the start and end of the numerically obtained diffusion profile (Figure 2b). This demands virtual identity of the analytically and numerically obtained values that always served as the first important control indicating that the numerical simulations were properly executed. We now use the *L*- and *R*-values to normalize the diffusion profiles: Equation (11) defines the diffusion influence *DI*. It goes from 0 or 0%, when *R*_eff_ approaches *L*, to 1 or 100%, when *R*_eff_ approaches *R* (Figure 2c). Thus, we distinguish in Figure 2 between the diffusion profiles, the *R*_eff_(*D*) or *R*_eff_(*D*^1/2^) profiles (Figure 2b), and the diffusion-influence profiles, the *DI*(*D*) or *DI*(*D*^1/2^) profiles (Figure 2c), or the *DI*(*J*), *DI*(*J*^1/2^), or *DI*(*X*) profiles (Figure 2d) after we discovered that the decisive independent variable, the decisive x-variable, is not *D* but *J* or its square root, *X* (Equation (12)).

(11) $$DI=\frac{R_{\mathrm{eff}}-L}{R-L}$$

In our quest for the HSJE, for an analytic equation that captures all possible diffusion profiles, we systematically varied the initial distance distribution as well as all coefficients of the HSE. Doing that, we found that, as mentioned, the appropriate independent variable is not the diffusion coefficient, *D*, but what we now call the augmented diffusion coefficient, *J*, composed of three coefficients: the diffusion coefficient, the donor lifetime, and the Förster radius (Equation (12)). Remarkably, *J* is dimensionless. To repeat, we address plots of *DI* versus *J* or its square root, *X* = *J*^1/2^, as diffusion-influence profiles, as *DI*, *DI*(*J*), or *DI*(*X*) profiles.

(12) $$J=\frac{D\tau_{D}}{R_{0}^{2}};\;X=\sqrt{J}=\frac{\sqrt{D\tau_{D}}}{R_{0}}$$

We found that all *DI*-profiles are sigmoids that can be fitted to Equation (13), where *X*^0^ = *J*^1/2^. The value *X*^0^ is the *X* value where *DI* = 50%; the *M* value describes the steepness of the sigmoid.

(13) $$DI\left(X\right)=\frac{{\left(X/X_{0}\right)}^{M}}{1+{\left(X/X_{0}\right)}^{M}}$$

The sigmoidal profiles obtained from the HSE solutions appear to be perfectly point symmetrical around the point (*X*_0_, 50%) provided that a logarithmic scale is chosen for the x-axes or, equivalently, that *DI* is plotted against ln*X*. The numerically obtained profiles could then be perfectly fitted according to Equation (13). Thus, we are convinced that any *DI* profile follows a symmetrical sigmoid in accordance with Equation (13). This requirement of sigmoidal symmetry served as the second important control that indicates correctly performed numerical simulations. Equations (12) and (13), in combination, yield Equation (14), a first raw form of the HSJE.

In the course of our investigation, we discovered and formulated four equivalence statements that led us from *D* as the independent variable in the diffusion profiles, to *J* or its square root, *X*, as independent variables in the diffusion-influence profiles. We learned that the parameters *X*_0_ and *M* in Equation (13) are functions of just a single variable, of the ratio of the Förster radius and the left-integration limit, of *R*_0_/*r*_L_. Thus, in the last step, we completed the HSJE by numerical work to determine how *X*_0_ and *M* depend on *R*_0_/*r*_L_.

(14) $$R_{\mathrm{eff}}=\frac{{\left(X/X_{0}\right)}^{M}}{1+{\left(X/X_{0}\right)}^{M}}\cdot \left(R-L\right)+L$$

### 3.2. The Equivalence Statements

In the following, we always compare effective-distance and diffusion-influence values and profiles obtained from the HSE used with two different sets of inputs that we distinguish by (1) and (2) as superscript. That is, we compare HSE^(1)^ and HSE^(2)^, where HSE^(1)^ uses set^(1)^ that consists of the distance distribution, *p*^(1)^(*r*), and the values, *D*^(1)^, *τ*_D_^(1)^, *R*_0_^(1)^, and *r*_L_^(1)^, while HSE^(2)^ uses set^(2)^ that consists of the distance distribution, *p*^(2)^(*r*), and the values, *D*^(2)^, *τ*_D_^(2)^, *R*_0_^(2)^, and *r*_L_^(2)^. Accordingly, the augmented diffusion coefficient *J*^(1)^ equals *D*^(1)^*τ*_D_^(1)^/(*R*_0_^(1)^)^2^ and *J*^(2)^ equals *D*^(2)^*τ*_D_^(2)^/(*R*_0_^(2)^)^2^. We also have to distinguish between the two diffusion-influence profiles obtained from HSE^(1)^ and HSE^(2)^, between *DI*^(1)^ and *DI*^(2)^ as functions of *J* or *X*, as they usually stem from different distance distributions, *p*^(1)^(*r*) and *p*^(2)^(*r*), that usually come with different *L*- and *R*-values, *L*^(1)^ and *R*^(1)^ and *L*^(2)^ and *R*^(2)^ (see Equations (9)–(11)).

#### 3.2.1. The First Equivalence Statement: Variation of the Distance Distribution

**Figure 3:**
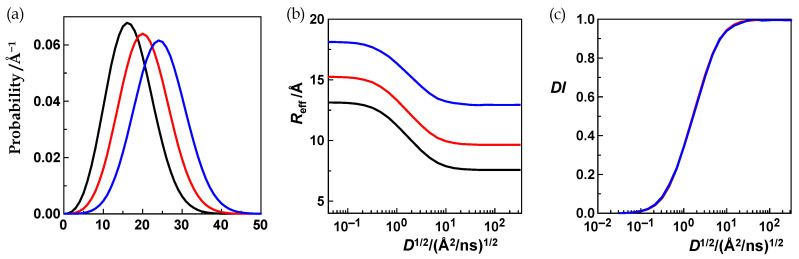
(**a**) Three different 3-D Gaussian distance distributions (black, red, blue). (**b**) The corresponding diffusion profiles (black, red, blue) with the effective distance plotted against the square root of the diffusion coefficient. The donor lifetime, the Förster radius, and the left integration limit were kept constant (*τ*_D_ = 100 ns, *R*_0_ = 10 Å, *r*_L_ = 2.5 Å) (**c**) After normalization (Equation (11)), the three profiles became identical.

To expose what happens if we only vary the distance distribution, we have chosen the three distributions shown in Figure 3a. The diffusion profiles that correspond to these distributions and to the chosen photophysical parameters are given in Figure 3b. These profiles are expectedly very different because any specific distance distribution comes with specific *L*- and *R*-values (Equations (9) and (10)). However, when we calculate the *DI*-values by using the *L*- and *R*-values characteristic for each distribution (Equation (11)), and normalize the profiles that way, we obtain perfect overlap of the resulting diffusion-influence profiles (Figure 3c). This normalization, the introduction of the diffusion-influence or *DI*-value defined by Equation 11 opened our path towards the HSJE.

We now express the first equivalence statement (ES1) as text and, then, in formula.

##### Equivalence Statement 1 (ES1, Text)

“The diffusion-influence profiles of any two well-behaved probability distributions, *p*^(1)^(*r*) and *p*^(2)^(*r*), coincide if they were obtained with the same values of the donor lifetime, *τ*_D_, the Förster radius, *R*_0_, and the left-integration limit, *r*_L_.”

*ES1 (formal):*

(15) $$\begin{array}{l} \mathrm{For}\;\mathrm{any}\;p^{\left(1\right)}\left(r\right)\;\mathrm{and}\;p^{\left(2\right)}\left(r\right):\;\mathrm{If}\;D^{\left(1\right)}=D^{\left(2\right)},\;\tau_{D}^{\left(1\right)}=\tau_{D}^{\left(2\right)},\;R_{0}^{\left(1\right)}=R_{0}^{\left(2\right)},\;\mathrm{and}\;r_{L}^{\left(1\right)}=r_{L}^{\left(2\right)}\\ \mathrm{then}\;DI^{\left(1\right)}\left(J^{\left(1\right)}\right)=DI^{\left(2\right)}\left(J^{\left(2\right)}\right) \end{array}$$

Here and in the three following statements, we always use the augmented diffusion coefficient, *J*, defined by Equation (12), as the independent variable in diffusion-influence or *DI* profiles. In this first statement, *J*^(1)^ and *J*^(2)^ are obviously identical, and using *J* as the independent variable makes no difference versus using *D* instead, but it fosters the formal parallelism of the four equivalence statements. Two questions arise:

1. Why do we demand that *r*_L_^(1)^ = *r*_L_^(2)^? Why can ES1 not be applied if the left-integration limit differs in both sets?

In case of a smaller left-integration value, the contribution of diffusion to FRET at infinite diffusion is higher. This can be concluded from term-(2) of the HSE (Equation (5)) that encompasses Förster’s law (Equation (1)): At smaller *r*_L_ values, FRET becomes faster. As a consequence, higher values of the augmented diffusion coefficient are necessary to reach the dynamic limit in the diffusion-influence profiles. Therefore, the distribution with smaller *r*_L_ will lead to a diffusion-influence profile that only later reaches *DI* values close to 100%. This is further detailed and illustrated in the Supporting Information, Chapter 7b, Figure S4a–c.

2. Is ES1 valid for any distance distribution equation or model? Or is it only valid for “well-behaved” models?

Prominent model equations that have been chosen in the past to describe end-to-end distance distributions in flexible peptides have been the one-parameter ideal-chain or random-coil model (Equation (16), parameter *b*) or the two-parameter 3D-Gaussian or skewed-Gaussian model (Equation (17), parameters *a* and *b*). The normalization constant, *N*, is determined through the normalization condition: The integral over *p*(*r*) from left to right integration limit has to be unity (see the Supporting Information, Chapter 7c, Equation (S26)).

(16) $$p(r)=Nr^{2}\cdot \left(\frac{3}{2\pi b^{2}}\right)\cdot \exp\left(-\frac{3r^{2}}{2b^{2}}\right);\;b={\langle r^{2}\rangle }^{1/2}$$

(17) $$p(r)=Nr^{2}\cdot \exp\left(-a\left(b-r^{2}\right)\right)$$

Using Equations (16) or (17), we discovered that sometimes the *DI*-overlap of the profiles of two such distributions became less perfect in case of large discontinuities of the distributions at the point *r* = *r*_L_, that is, in the case of large jumps of probability-density values from zero to finite values at this point (Supporting Information, Chapter 7c, Figure S5a). In the transition region of the sigmoidal *DI*-profiles of two such distributions, the *DI*-value disagreement can become as large as 10%, undermining our efforts to develop a closed analytic equation with precisely determined constants, the HSJE. Further, such discontinuities can hardly be justified by any physical reasoning. This problem was resolved, and complete overlap was restored for all cases when we modified the distribution equations to display continuity of probability-density values at and around *r* = *r*_L_ (Equations (18) and (19)), (Supporting Information, Chapter 7c, Figure S5b). Thus, we proceeded by using only “well-behaved” distribution equations that, by their very nature, guarantee that *p*(*r* = *r*_L_) = 0 and guarantee *p*(*r*)-value continuity (Equations (18) and (19)).

(18) $$p(r)=Nr_{0}^{2}\cdot \left(\frac{3}{2\pi b^{2}}\right)\cdot \exp\left(-\frac{3r_{0}^{2}}{2b^{2}}\right);\;r_{0}=r-r_{L}$$

(19) $$p(r)=Nr_{0}^{2}\cdot \exp\left(-a\left(b-r_{0}^{2}\right)\right);\;r_{0}=r-r_{L}$$

#### 3.2.2. The Second Equivalence Statement: Variation of the Donor Lifetime

**Figure 4:**
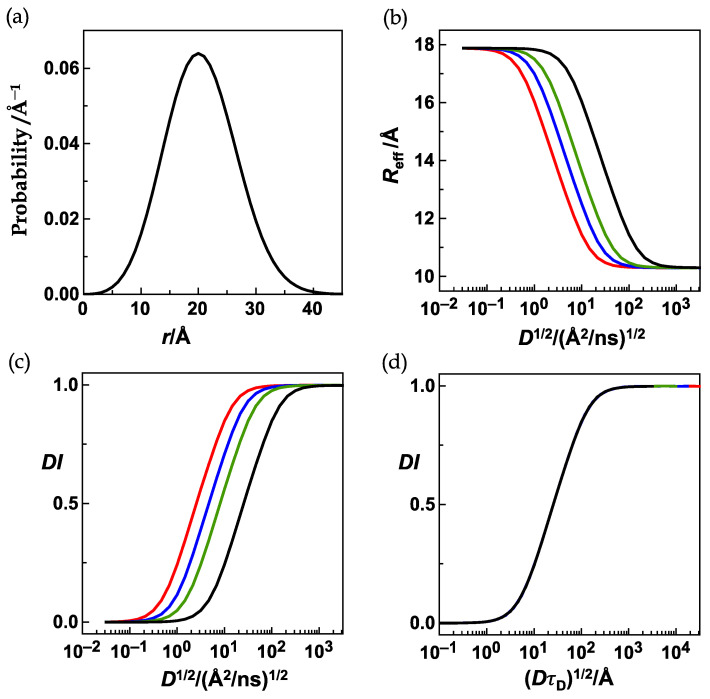
(**a**) A 3-D Gaussian distance distribution (**b**) Four diffusion profiles obtained with (**a**) four different donor lifetime constants, *τ*_D_, of 100 ns (red), of 30 ns (blue), of 10 ns (green), and of 1 ns (black). The Förster radius (*R*_0_ = 15 Å) and the left integration limit (*r*_L_ = 3 Å) were held constant. (**c**) Diffusion-influence profiles after normalization. (**d**) The *DI*-profiles coincide when the *DI* values are plotted against the square root of the product of diffusion coefficient and donor lifetime.

Next, we vary the donor lifetime. Figure 4a shows an exemplary distance distribution, and Figure 4b the four diffusion profiles that were obtained for this distribution and for the four donor lifetime values, 100 ns (red curve, left), 30 ns (blue curve), 10 ns (green curve), and 1 ns (black curve, right). All other parameters of the HSE were kept constant. Using the same color code, Figure 4c shows the corresponding diffusion-influence profiles. When we now plot, for each profile, the diffusion-influence value against the product of the diffusion coefficient and the corresponding donor lifetime, 100, 30, 10, or 1 ns, or against the square root of this product, we observe that the four profiles become identical (Figure 4d). Going from the diffusion coefficient, *D*, to the product *D*⋅*τ*_D_ as the independent variable in diffusion-influence plots is the first of two steps towards the augmented diffusion coefficient, *J*.

This observed profile identity means that the values of the effective distance obtained from the HSE with input sets, set^(1)^ and set^(2)^, that only differ by the diffusion coefficient, *D*^(1)^ in contrast to *D*^(2)^, and by the donor lifetime, *τ*_D_^(1)^ in contrast to *τ*_D_^(2)^, are identical as long as the product *D*⋅*τ*_D_ does not change, that is, as long as it is valid that *D*^(1)^*τ*_D_^(1)^ = *D*^(2)^*τ*_D_^(2)^. We now formulate the second equivalence statement and prove it to be a mathematical consequence of the HSE.

##### Equivalence Statement 2 (ES2, Text)

“Given any probability distribution: If the diffusion coefficient is varied from *D*^(1)^ to *D*^(2)^ and the donor lifetime from *τ*_D_^(1)^ to *τ*_D_^(2)^, but the product of diffusion coefficient and donor lifetime remains constant, then the diffusion-influence values obtained from the diffusion-influence functions, *DI*^(1)^(*J*^(1)^) and *DI*^(2)^(*J*^(2)^), are identical. The corresponding diffusion-influence profiles coincide”.

*ES2 (formal):*

(20) $$\begin{array}{l} \mathrm{For}\;\mathrm{any}\;p^{\left(1\right)}\left(r\right)=p^{\left(2\right)}\left(r\right):\;\mathrm{If}\;D^{\left(1\right)}\tau_{D}^{\left(1\right)}=D^{\left(2\right)}\tau_{D}^{\left(2\right)},\;R_{0}^{\left(1\right)}=R_{0}^{\left(2\right)},\;\mathrm{and}\;r_{L}^{\left(1\right)}=r_{L}^{\left(2\right)}\\ \mathrm{then}\;DI^{\left(1\right)}\left(J^{\left(1\right)}\right)=DI^{\left(2\right)}\left(J^{\left(2\right)}\right) \end{array}$$

ES2 can be derived in several ways from the HSE (Equation (5)), as detailed in the Supporting Information, Chapter 7d.

Here we opted for a change-of-variable approach applied to the HSE (Equation (5)). We use the fact that any rescaling of time can impossibly affect the value of the effective distance (see Supporting Information, Chapter 7d). We rescale the time with the donor lifetime (Equation (21)) and insert the resulting substitutions into the HSE to arrive at Equation (22) that simplifies to Equation (23).

(21) $$z=\frac{t}{\tau_{D}}\;\to \;\partial z=\frac{\partial t}{\tau_{D}}\;\to \;\partial t=\tau_{D}\partial z$$

(22) $$\frac{1}{\tau_{D}}\frac{\partial N\left(r,z\right)}{\partial t}=-\frac{1}{\tau_{D}}N\left(r,z\right)-\frac{1}{\tau_{D}}\frac{R_{0}^{6}}{r^{6}}N\left(r,z\right)+D\frac{\partial }{\partial r}\left(N_{0}\left(r\right)\frac{\partial \left(N/N_{0}\right)}{\partial r}\right)$$

(23) $$\frac{\partial N\left(r,z\right)}{\partial z}=-N\left(r,z\right)-\frac{R_{0}^{6}}{r^{6}}N\left(r,z\right)+D\tau_{D}\frac{\partial }{\partial r}\left(N_{0}\left(r\right)\frac{\partial \left(N/N_{0}\right)}{\partial r}\right)$$

We observe that the product of diffusion coefficient and donor lifetime, *D*⋅*τ*_D_, appears in the rescaled HSE (Equation (23)) at the same place as *D* does in the initial HSE (Equation (5)). Secondly, neither *D* nor *τ*_D_ appear at any other place of the rescaled HSE (Equation (23)). It is obvious from Equation (23) that any change of the diffusion coefficient and donor lifetime will not change the resulting effective distance, *R*_eff_, if their product, *D*⋅*τ*_D_, does not change. This is what ES2 expresses.

#### 3.2.3. The Third Equivalence Statement: Variation of the Förster Radius and Left-Integration Limit

In the next step, we only change the Förster radius from *R*_0_^(1)^ to *R*_0_^(2)^ and the left-integration value from *r*_L_^(1)^ to *r*_L_^(2)^. We observe total overlap of the resulting diffusion-influence profiles, *DI*^(1)^(*J*^(1)^) and *DI*^(2)^(*J*^(2)^), if the third equivalence statement is obeyed:

##### Equivalence Statement 3 (ES3, Text)

“Given any probability distribution: If the Förster radius and the left-integration limit are varied but not their ratio, then the diffusion-influence values obtained from the diffusion-influence functions, *DI*^(1)^(*J*^(1)^) and *DI*^(2)^(*J*^(2)^), are identical. The corresponding diffusion-influence profiles coincide”.

*ES3 (formal):*

(24) $$\begin{array}{l} \mathrm{For}\;\mathrm{any}\;p^{\left(1\right)}\left(r\right)=p^{\left(2\right)}\left(r\right):\;\mathrm{If}\;\frac{D^{\left(1\right)}\tau_{D}^{\left(1\right)}}{{\left(R_{0}^{\left(1\right)}\right)}^{2}}=\frac{D^{\left(2\right)}\tau_{D}^{\left(2\right)}}{{\left(R_{0}^{\left(2\right)}\right)}^{2}}\;\mathrm{and}\;\frac{R_{0}^{\left(1\right)}}{r_{L}^{\left(1\right)}}=\frac{R_{0}^{\left(2\right)}}{r_{L}^{\left(2\right)}}\\ \mathrm{then}\;DI^{\left(1\right)}\left(J^{\left(1\right)}\right)=DI^{\left(2\right)}\left(J^{\left(2\right)}\right) \end{array}$$

**Figure 5:**
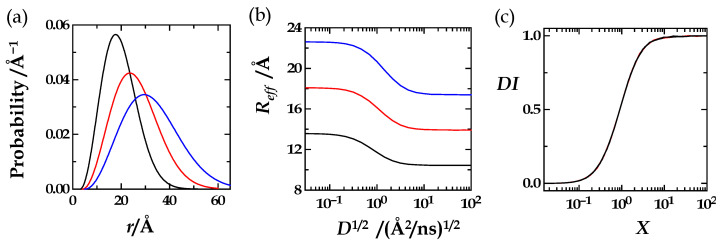
(**a**) Three ideal-chain distance distributions (Equation (18)) with *b* = 18 Å, *r*_L_ = 3 Å (black curve); *b* = 24 Å, *r*_L_ = 4 Å (red curve); and *b* = 30 Å, *r*_L_ = 5 Å (blue curve). (**b**) The corresponding diffusion profiles obtained with *b* = 18 Å, *r*_L_ = 3 Å, *R*_0_ = 9 Å (black curve); with *b* = 24 Å, *r*_L_ = 4 Å, *R*_0_ = 12 Å (red curve); and with *b* = 30 Å, *r*_L_ = 5 Å, *R*_0_ = 15 Å (blue curve). Thus, for all three evaluated distributions (black, red, blue), the ratio *R*_0_/*r*_L_ equaled 3. (**c**) The corresponding diffusion-influence profiles with *DI* plotted against *X* (*X* = *J*^1/2^). The three profiles merge into one.

That ES3 holds true with excellent precision is illustrated in Figure 5. Here, we also tested ES1 again: We compared three different distance distributions and their *DI*-profiles obtained with three different pairs of *R*_0_-and *r*_L_-values where *R*_0_/*r*_L_ was always held constant at 3. The three corresponding *DI*-profiles coincide (Figure 5c, *X* = *J*^1/2^).

To explain ES3, we continue to use the change-of-variable approach. After rescaling time with the donor lifetime, we now rescale space with the Förster radius. The new space coordinate is defined by Equation (25).

(25) $$s=\frac{r}{R_{0}}\;\to \;\partial s=\frac{\partial r}{R_{0}}\;\to \;\partial r=R_{0}\partial s$$

How term-(2) of the HSE (Equation (5)) changes after time rescaling and subsequent space rescaling is shown in Equation (26).

(26) $$-\frac{1}{\tau_{D}}{\left(\frac{R_{0}}{r}\right)}^{6}N(r,t)=-{\left(\frac{R_{0}}{r}\right)}^{6}N(r,z)=-\frac{1}{{\left(\frac{r}{R_{0}}\right)}^{6}}N(r,z)=-\frac{1}{s^{6}}N(s,z)$$

Thus, the HSE, now written not with the old (*r*,*t*)-coordinates but with the new (*s*,*z*)-coordinates, is expressed by Equation (27) or, when we insert the definition of the augmented diffusion coefficient, *J*, (Equation (12)), by Equation (28).

(27) $$\frac{\partial N\left(s,z\right)}{\partial z}=-N\left(s,z\right)-\frac{1}{s^{6}}N\left(s,z\right)+\frac{D\tau_{D}}{R_{0}^{2}}\frac{\partial }{\partial s}\left(N_{0}\left(s\right)\frac{\partial \left(N/N_{0}\right)}{\partial s}\right)$$

(28) $$\frac{\partial N\left(s,z\right)}{\partial z}=-N\left(s,z\right)-\frac{1}{s^{6}}N\left(s,z\right)+J\frac{\partial }{\partial s}\left(N_{0}\left(s\right)\frac{\partial \left(N/N_{0}\right)}{\partial s}\right)$$

**Figure 6:**
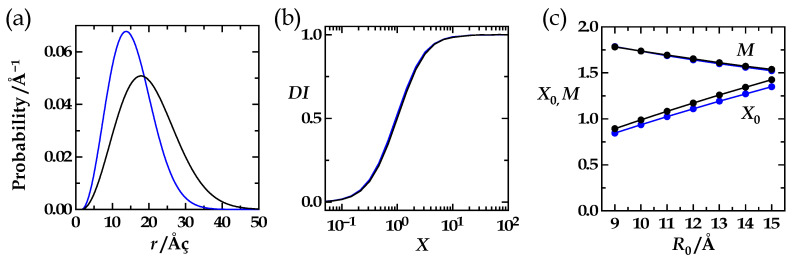
The HSE was solved for two different series of ideal-chain distributions (Equation (18)). *R*_0_ was varied from 9 Å to 15 Å, and *r*_L_ was varied from 1.5 Å to 5 Å. The donor lifetime was held constant (*τ*_D_ = 100 ns). In one series of distributions, the constant *b* was chosen as *b* = 1.5⋅*R*_0_ ranging from 13.5 Å to 22.5 Å, in the other series as *b* = 2⋅*R*_0_ ranging from 18 Å to 30 Å. (**a**) Exemplary distributions for the two series with *r*_L_ = 3 Å, *R*_0_ = 10 Å and either *b* = 1.5⋅*R*_0_ = 15 Å (blue curve) or *b* = 2⋅*R*_0_ = 20 Å (black curve). (**b**) The *DI*(*X*) profiles overlap for both distributions shown in (**a**). (**c**) All *DI*(*X*) profiles were analyzed by using Equation (13).

When we rescale space, we also rescale the distance distribution, and that also means that we rescale the integration limits of the distance distribution. As discussed, changing the left-integration limit has a strong effect on the diffusion-influence profile. We need to pay less attention to the right-integration limit, *r*_R_, as simulations showed that *r*_R_ has only to be chosen to be sufficiently large to enclose the distance distribution up to probability-density values close to zero. Additionally, the changing shape of the distribution caused by rescaling, its expansion or contraction in the x-direction, is of no consequence, as has been demonstrated (ES1, Figure 3 and Figure 6). Nevertheless, we keep in mind, in the ongoing analysis, the validity of ES3 rests on the validity of ES1. After rescaling, in accordance with Equation (26), the new left-integration limit follows Equation (29).

(29) $$s_{L}=\frac{r_{L}}{R_{0}}$$

Thus, when we change *R*_0_^(1)^ to *R*_0_^(2)^ and *r*_L_^(1)^ to *r*_L_^(2)^ and apply space rescaling to the corresponding HSE equations, HSE^(1)^ and HSE^(2)^, we obtain *J*^(1)^ and *J*^(2)^ as well as *s*_L_^(1)^ and *s*_L_^(2)^. Only if *s*_L_^(1)^ and *s*_L_^(2)^ are identical can we expect that the diffusion-influence values *DI*^(1)^(*J*^(1)^) and *DI*^(2)^(*J*^(2)^) and corresponding profiles coincide. According to Equation (29), this will be the case if *r*_L_^(1)^/*R*_0_^(1)^ = *r*_L_^(2)^/*R*_0_^(2)^. This is equivalent to the condition used in ES3: *R*_0_^(1)^/*r*_L_^(1)^ = *R*_0_^(2)^/*r*_L_^(2)^.

#### 3.2.4. The Fourth Equivalence Statement

Taken together, the three established statements combine into a fourth one.

##### Equivalence Statement 4 (ES4, Text)

“For any pair of distance distributions, *p*^(1)^(*r*) and *p*^(2)^(*r*): If the diffusion coefficient or the donor lifetime or the Förster radius are varied but not the augmented diffusion coefficient *J* composed of these parameters, and if the ratio of the Förster radius and left-integration limit is not varied, then the diffusion-influence values obtained from the diffusion-influence functions, *DI*^(1)^(*J*^(1)^) and *DI*^(2)^(*J*^(2)^), are identical. The corresponding diffusion-influence profiles coincide.”

*ES4* (*formal*):

(30) $$\begin{array}{l} \mathrm{For}\;\mathrm{any}\;p^{\left(1\right)}\left(r\right)\;\mathrm{and}\;p^{\left(2\right)}\left(r\right):\;\mathrm{If}\;\frac{D^{\left(1\right)}\tau_{D}^{\left(1\right)}}{{\left(R_{0}^{\left(1\right)}\right)}^{2}}=\frac{D^{\left(2\right)}\tau_{D}^{\left(2\right)}}{{\left(R_{0}^{\left(2\right)}\right)}^{2}}\;\mathrm{and}\;\frac{R_{0}^{\left(1\right)}}{r_{L}^{\left(1\right)}}=\frac{R_{0}^{\left(2\right)}}{r_{L}^{\left(2\right)}}\\ \mathrm{then}\;DI^{\left(1\right)}\left(J^{\left(1\right)}\right)=DI^{\left(2\right)}\left(J^{\left(2\right)}\right) \end{array}$$

This means that a fixed *R*_0_/*r*_L_ ratio corresponds to a single sigmoidal curve, a single diffusion-influence profile. We slide along that sigmoid when we vary *J*, that is (see Equation (11)), when we vary the diffusion coefficient, or the donor lifetime, or the Förster radius and the left-integration limit, but latter two only in combinations that keep *R*_0_/*r*_L_ constant. Such a sigmoid follows Equation (13) with constant coefficients, *X*_0_ and *M*.

Thus, if the *R*_0_/*r*_L_ ratio is fixed, *X*_0_ and *M* are constants that can be determined by fitting the numerically obtained sigmoid to Equation (13). As soon as we vary *R*_0_/*r*_L_, we obtain a different sigmoid characterized by different *X*_0_- and *M*-values. Thus, *X*_0_- and *M* are functions of *R*_0_/*r*_L_. We need to determine these functions to establish Equation (13) as an equation that covers all possible diffusion-influence profiles. Being content to firstly cover only a small two-dimensional range of *R*_0_ and *r*_L_, we learned that these functions are then simple polynomials of second degree. In the following, the focus shifts from mainly mathematical reasoning to the need to obtain reliable results from numerical HSE-solutions.

### 3.3. The Grid

We determined these polynomial functions firstly for a limited two-dimensional range of 9–15 Å for *R*_0_ and of 1.5–5 Å for *r*_L_: These are the crucial *R*_0_- and *r*_L_-ranges for short-distance FRET methods applied to flexible peptides or polymers [13,17,18] (see Supporting Information, Chapter 3, Figures S1 and S2). The *R*_0_,*r*_L_-grid, or *R*_0_,*r*_L_-set, was *R*_0_/Å × *r*_L_/Å = {9.0, 10.0, 11.0, 12.0, 13.0, 14.0, 15.0} × {1.5, 2.0, 2.5, 3.0, 3.5, 4,0, 4.5, 5.0} resulting in a total of 56 *R*_0_,*r*_L_-combination. For each combination, we determined the corresponding *DI*-profile by numerically solving the HSE for at least 28 values of the diffusion coefficient. We then determined the *X*_0_- and *M*-value of each of these 56 profiles by fitting them to Equation (13). Indeed, the *X*_0_- and *M*-values coincided for all those *R*_0_,*r*_L_-combinations with a constant ratio *R*_0_/*r*_L_. A selection of results, those for *R*_0_/*r*_L_ equal to 3, 4, 5, or 6, are shown in Table 1. These results clearly corroborate ES3 and, by that, also ES1, on which ES3 rests.

Which distance distributions were used in this analysis? To obtain highly reliable *X*_0_- and *M*-values from fits of the numerically obtained sigmoids required perfectly symmetrical sigmoids in the first place. Recall, that we argued in the first chapter of Results, “Ideas and Concepts”, that any diffusion-influence profile should be a perfectly symmetrical sigmoid, a condition that when met points to a properly executed numerical simulation. A consequence of the normalization Equation (11) is that sigmoids numerically obtained from the HSE follow Equation (13) less accurately when the difference between the extreme values of the effective distance, *L* and *R*, when the amplitude of the sigmoid, ∆*R*_eff_ = *L* − *R*, becomes smaller. Very narrow distance distributions only allow for accordingly small ∆*R*_eff_-values. It is then almost impossible to numerically obtain well-shaped sigmoids. Luckily, we were free to choose the distance distributions in any way we wanted (ES1) to evaluate the grid. We have chosen the simplest model possible, the ideal-chain or random coil model (Equation (18)). Here, the broadness of the distribution is determined by the parameter, *b*. Thus, to always guarantee broad distributions and to always guarantee a large difference, ∆*R*_eff_ = *L* − *R*, when *R*_0_ is varied within the grid, the parameter *b* was chosen to follow the equation *b* = 2⋅*R*_0_. An exemplary distribution (*R*_0_ = 10 Å, *r*_L_ = 3 Å) of this series is given by the black curve in Figure 6a.

We performed the same analysis with a series of less broad distributions following *b* = *R*_0_ and *b* = 1.5⋅*R*_0_. An exemplary distribution (*R*_0_ = 10 Å, *r*_L_ = 3 Å) of the latter is given by the blue curve in Figure 6a. Figure 6b compares the diffusion-influence profiles for *R*_0_ = 10 Å, *r*_L_ = 3 Å, *b* = 2⋅*R*_0_ (black curve) and for *R*_0_ = 10 Å, *r*_L_ = 3 Å, *b* = 1.5⋅*R*_0_ (blue curve). Both profiles overlap so well that visual distinction is impossible. This is valid for comparisons at any of the employed *R*_0_-values as is shown in Figure 6c. The *M*-values were virtually identical, and the *X*_0_-values (lower black and blue data points) were always so close that the corresponding *DI*-profiles were visually indistinguishable (see Figure 6b).

This comparative analysis confirmed that distributions with *b* = 2⋅*R*_0_ are sufficiently broad to guarantee accurate numerical results. In addition, at this point, we have gained strong confidence in the validity of ES1 and ES3 and in all of the equivalence statements.

The figure illustrates how *X*_0_ and *M* vary with *R*_0_ for the two series when *r*_L_ is kept at 3 Å. For *M*, the values obtained for the two series overlap (upper black and blue circles); for *X*_0_, the values (lower black and blue circles) obtained for the two series are so close that the corresponding diffusion profiles (see panel b) are visually indistinguishable.

### 3.4. The HSJE

**Figure 7:**
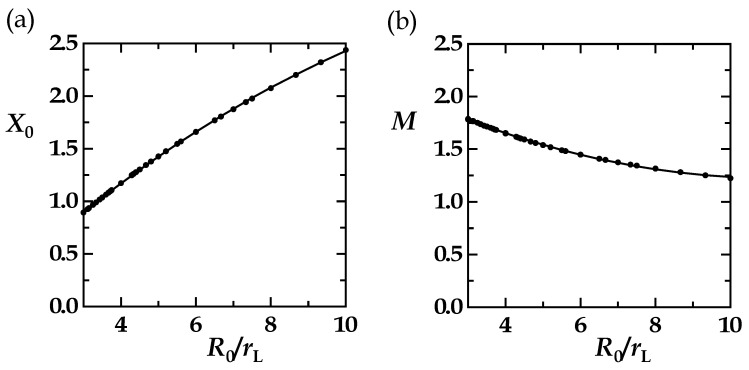
The results for *X*_0_ and *M* (solid circles in panels (**a**,**b**) obtained for the range of *R*_0_ = 9–15 Å, *r*_L_ = 1.5–5 Å with *b* = 2*R*_0_, and *R*_0_/*r*_L_ > 3) were fitted to second-degree polynomial functions (solid lines in panels (**a**,**b**) given in Table 2).

We can now analyze the results obtained for *X*_0_ and *M* for the distribution series with *b* = 2*R*_0_, the series that guarantees highest numerical accuracy by leading to the largest ∆*R*_eff_ = *L* − *R* differences or *DI*(*X*) amplitudes. We obtained smooth curves that could be fitted to second-degree polynomial functions. Data points and fitting curves coincided (Figure 7). The functions and coefficients are given in Table 2.

**Figure 8:**
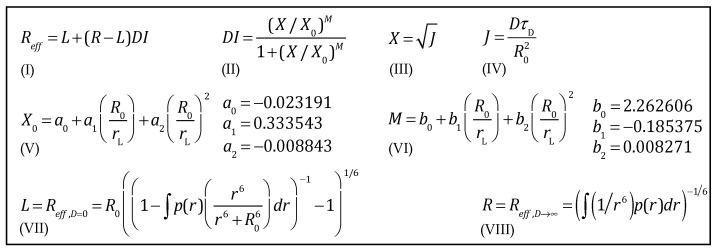
The Haas-Steinberg–Jacob equation (HSJE) is a closed analytical equation decomposed here into simple equations, I to VIII, for clarity. With the coefficients shown here (*a*_0_ to *b*_3_), it is valid for *R*_0_ 9–15 Å and *r*_L_ 1.5–5 Å.

We are ready to state the HSJE for the given range of *R*_0_ = 9–15 Å and *r*_L_ = 1.5–5 Å (see the legend of Figure 7) in Figure 8. For clarity, we have decomposed the HSJE into its simple constituents, equations (I) to (VIII). The HSJE written as a single equation is shown in Supporting Information, Chapter 7e.

We now have a closed analytical expression, a CAE, for a defined parameter range. As with any other equation, it can now be applied to global analyses. The preliminary insights gained from such applications are very interesting but not the main topic of this article. We therefore referred these first applications of the HSJE to the Supporting Information, Chapter 8, Figures S6a–d and S7a–d.

## 4. Discussion

We have followed the full path from a partial differential equation, the HSE, to a closed analytical expression, the HSJE. Its benefits are of a theoretical but also of a mere practical nature: The HSJE is usually solved in seconds, not in minutes or hours, by two orders of magnitude faster than the HSE. This advantage amplifies when the HSJE is used as the basis of a global analysis that generally requires numerous fitting cycles to reach the best values for the diffusion coefficient, *D*; the distance distribution, *p*(*r*); and the left-integration limit, *r*_L_. Experimental values of the effective distance can be obtained from time-resolved fluorescence measurements but equally well from more widely available steady-state fluorescence spectroscopy. The HSJE is certainly not trivial but is still simple enough that multivariate or global analysis can be performed in commonly available programs such as Microsoft Excel.

DFRET is just one method in a whole range of optical methods for studying the structure and dynamics of polymers [10,11,12,13,14,17,19,20,21,22,23,24,25,26,27,28,29,30,31,32,33,34,35,36,37,38,39,40,41,42,43,44,45,46,47,48,49,50,51,52,53,54,55,56,57,58,59,60,61,62]. When we go into detail, we realize that any progress in DFRET can synergistically promote progress in this entire field [13]. The structure and dynamics of polymers are so important that numerous methods based on two optical molecular labels have been developed [2,3,5,6,10,11,13,15,17,22,23,24,25,27,28,29,30,31,32,33,34,35,36,37,38,47,48,49,57,58,59,63,64,65,66,67,68]. These labels are either synthetically conjugated to the chain or naturally present, as, for instance, Trp or Tyr in a peptide or protein. One of the deepest motivations for developing such methods has always been the “protein folding problem”. The “static” folding problem now appears to have been solved by artificial intelligence methods applied to a huge amount of data [69] but countless questions about folding involving dynamics and mechanisms remain open. Examples include misfolding in amyloidosis diseases as well as the sometimes bizarre behavior of intrinsically disordered peptides and proteins [70].

These methods include not only FRET, but also CQ methods (collisional-quenching methods)1 [25,71]. For example, the sdFRET method is based on the excitation of a donor, e.g., the donor FTrp, in combination with Dbo as acceptor. The peculiarity of this FTrp/Dbo pair is that instead of FTrp, one can simply excite Dbo, whose fluorescence is then quenched by collision with FTrp [49]. This particular CQ method is called CIFQ (collision-induced fluorescence quenching). How quickly this quenching proceeds also informs the dynamics and flexibility of the chain. In the past, we have been able to reconcile seemingly contradictory results from different sdFRET, CIFQ, and CQ methods [13]. Therefore, we reiterate our assertion that any progress in DFRET or any of the other methods can synergistically promote progress in the entire field of polymer structure and dynamics. We believe that we have made such progress with the development of the HSJE.

The augmented diffusion coefficient. In addition to the practical benefits of the HSJE, we believe that its structure gives a more lucid insight into parameters whose variation can feed essential information into the needed global analysis. This is first of all the newly defined augmented diffusion coefficient, *J*. What does *J* actually mean? If we want to determine the diffusion impact and the diffusion coefficient, *D*, we need the widest possible range of the diffusion influence, *DI*, preferably from 0 to 100%. It is clear that any experimental series might cover a range within these limits but can never reach these limits themselves. We established that *DI* increases with *J* and that *J* increases linearly with *D* and *τ*_D_, the donor lifetime, and decreases with the square root of the Förster radius, *R*_0_ (Equation (18)). All of these variations have been employed in earlier attempts at a global analysis but without knowing which range of the diffusion influence was actually covered [3,7,9,10,14]. In this work, we obtained certainty that our previous variation [14] of the diffusion coefficient by solvent viscosity and variation of the donor lifetime by adding the viscogen ethylene glycol and by going from the donor FTrp to the donor NAla covered a *DI* range of 8 to 80% (comp. Supporting Information, Chapter 8c). In previous works [12,14], we also investigated and discussed the role of the donor quantum yield, and we continue this discussion in Supporting Information, Chapter 9, because the donor quantum yield can be made part of the HSJE as shown in Equations (S31)–(S33).

Preview. In summary, the diffusion contribution to FRET should not be neglected whenever we are not close to the static limit. Therefore, the question remains up to which *R*_0_-values and under which circumstances are we not close to this limit. Up to which *R*_0_-values should diffusion be included into the analysis, and up to which values should the HSJE be extended?

## 5. Conclusions

Fluorescence methods and methods based on fluorescence resonance energy transfer have asserted their place in questions of molecular/bio-molecular structure and dynamics simply because their speed and sensitivity are unsurpassed. We believe that also diffusion-enhanced Förster resonance energy transfer or DFRET has the potential to become influential, although the number of publications based on it has been small to date. DFRET ensemble analysis has been based on a partial differential equation, on the HSE. Using the HSE in a global analysis means that profound challenges must first be overcome. Furthermore, even with seemingly successful fits, doubts often remain about the realistic significance of the results obtained. By replacing the PDE with a closed analytical expression, by replacing the HSE with the HSJE, these difficulties can be ended.

## Figures and Tables

**Table 1:** Fitting results for *X*_0_ and *M* *.

*R*_0_/*r*_L_	*R*_0_	*r*_L_	*X*_0_	*M*
3	9	3	0.8935	1.7823
3	12	4	0.8929	1.7876
3	15	5	0.8933	1.7861
4	10	2.5	1.1722	1.6513
4	12	3	1.1719	1.6532
4	14	3.5	1.1724	1.6494
5	10	2	1.4259	1.5387
5	15	3	1.4256	1.5391
6	9	1.5	1.6591	1.4481
6	12	2	1.6592	1.4483
6	15	2.5	1.6587	1.4483

* The test distributions were chosen to follow Equation (18) with *b* = 2*R*_0_.

**Table 2:** *X*_0_ and *M* as second-degree polynomial functions of *R*_0_/*r*_L_.

*X*_0_ = *a*_0_ + *a*_1_ × (*R*_0_/*r*_L_) + *a*_2_ × (*R*_0_/*r*_L_)^2^; *M* = *b*_0_ + *b*_1_ × (*R*_0_/*r*_L_) + *b*_2_ × (*R*_0_/*r*_L_)^2^				
*n*	*X*_0_ =	*a_n_*-value	*M* =	*b_n_*-value
0	*a*_0_	−0.023191	*b*_0_	2.262606
1	*a*_1_ × (*R*_0_/*r*_L_)	0.333543	*b*_1_ × (*R*_0_/*r*_L_)	−0.185375
2	*a*_2_ × (*R*_0_/*r*_L_)^2^	−0.008843	*b*_2_ × (*R*_0_/*r*_L_)^2^	0.008271

The coefficients *a*_0_, *a*_1_, *a*_2_, and *b*_0_, *b*_1_, *b*_2_ are valid for the conditions *R*_0_ = 9–15 Å, *r*_L_ = 1.5–5 Å, *R*_0_/*r*_L_ > 3.

## Data Availability

Not applicable.

## Supplementary Materials

The following supporting information can be downloaded at: https://www.mdpi.com/article/10.3390/polym15030705/s1, The document “Supporting information: Diffusion-Enhanced Förster Resonance Energy Transfer in Flexible Peptides: From the Haas-Steinberg Partial Differential Equation to a Closed Analytical Expression”, which provides a detailed glossary, a review of relevant previously published information, an overview of basic concepts, aids to understanding the new findings, an initial application of the HSJE in a global analysis, and an introduction to ongoing discussion points.
