# Supplementary material for: Diffusion-Enhanced Förster Resonance Energy Transfer in Flexible Peptides: From the Haas-Steinberg Partial Differential Equation to a Closed Analytical Expression
Source: Polymers (Basel). 2023 Jan 30;15(3):705. doi: 10.3390/polym15030705 (PMC9919848; doi:10.3390/polym15030705)
Supplement: Supplementary file 1 [file polymers-15-00705-s001.zip › polymers-2140036-supplementary.pdf]

Supporting Information:

Diffusion-Enhanced Förster Resonance Energy Transfer in Flexible Peptides: From the Haas-Steinberg Partial Differential Equation to a Closed Analytical Expression

Maik H. Jacob,†, Roy N. D'Souza†, Alexandra I. Lazar†, Werner M. Nau*,†*

† School of Science, Constructor University, Bremen, Germany

**Corresponding authors*

e-mail:

M.H.J: m.jacob@jacobs-university.de

W.M.N: w.nau@jacobs-university.de

Table of Contents

1. Introduction
2. Symbols, Abbreviations, Glossary
3. How Experiments Lead to the Effective Distance
4. How Theory Leads to the Effective Distance
5. Fundamental Concepts
   - 5a. The Concept of the Energy Transfer Efficiency
   - 5b. The Concept of the Average
6. Extreme Effective Distances
   - 6a. The Effective Distance, *L*, in Absence of Diffusion
   - 6b. The Effective Distance, *R*, at Infinite Diffusion
7. Explanatory Notes on the Equivalence Statements
   - 7a. Survey of the Equivalence Statements
   - 7b. ES1: The Impact of the Left Integration Limit, r_L
   - 7c. ES1: "Well-Behaved Distance Distributions"
   - 7d. ES2: Variation of the Donor Lifetime
   - 7e. The HSJE Written as a Single Equation
8. Global Analysis
   - 8a. A Study with NAla sdFRET and FTrp sdFRET on a Flexible Peptide
   - 8b. Details of the Global Analysis Procedure
   - 8c. Applying the HSJE to Experimental Data in a Global Fit
9. Donor Quantum Yield
10. Mathematical Challenges
11. Outlook
12. References

1. Introduction

This Supporting Information file is closely aligned to the main text and is intended to enable the interested scientist to get a hold on every aspect of this article and to put the method to use.

2. Symbols, Abbreviations, Glossary

A	Acceptor.
A_D	Area under the kinetic trace of donor fluorescence deactivation in the donor-only peptide (Figure. S3 e,f and eq. S4).
A_{DA}	Area under the kinetic trace of donor fluorescence deactivation in the donor-acceptor peptide (Figure. S3 e,f and eq. S4).
a	The first of two parameters of a 3-D Gaussian distance probability distribution (eq. 17, eq. 19).
a_0, a_1, a_2	Coefficients obtained for the HSJE that describe the dependence of X_0 on the ratio R_0/r_l (Table 2, Fig. 8).
b	The second of two parameters of a 3-D Gaussian distance probability distribution (eq. 17, eq. 19).
b	The single parameter of the ideal-chain or random-coil distance distribution that determines its broadness (eq. 16, eq. 18)
b_0, b_1, b_2	Coefficients obtained for the HSJE that describe the dependence of M on the ratio R_0/r_L (Table 2, Fig. 8).
CAE	Closed Analytical Expression.
D	Diffusion coefficient (Font *italics*) measured in units of Å^2/ns in this ms.
D	Donor (Font regular).
D-peptide	Donor-only peptide that carries no acceptor.
DA-peptide	Donor-acceptor peptide.
DBO	2,3-diazabicyclo[2.2.2]oct-2-ene (Fig. S1).
Dbo	2,3-diazabicyclo[2.2.2]oct-2-ene (DBO) coupled to an asparagine. residue; FRET acceptor that enables sdFRET measurements (Fig. S1).

DI	The diffusion influence ranging from 0 to 100% (eq. 11).
DI(X)-profile	The dependency of the diffusion influence on the square root of the augmented diffusion coefficient, $J^{1/2}=X$ (eq. 12, eq. 13).
E	The energy transfer efficiency ranging from 0 to 100% (eq. S3-5, eq. S6a, eq. S7, eq. S9a, Chapter 5a
ES1-4	The equivalence statements developed in the main text and surveyed in the SI, Chapter 7.
ES1	*DI(X)* profiles do not depend on the distance distribution
ES2	*DI(X)* profiles coincide if the product of diffusion coefficient and donor lifetime is the same for the compared profiles and nothing else has changed.
ES3	*DI(X)* profiles coincide if the ratio of Förster radius and left integration limit are the same for the chosen distribution and nothing else has changed.
ES4	*DI(X)* profiles coincide if the ratio of Förster radius and left integration limit are the same for the chosen distributions.
FRET	Förster resonance energy transfer.
FTrp	5-L-Fluorotryptophan; it is used as a donor and displays monoexponential fluorescence kinetics after excitation (Fig. S1, Fig S2b).
FTrp sdFRET	A short-distance FRET method that employs the donor-acceptor pair, 5-L- Fluorotryptophan and Dbo. The Förster radius, R_0, is only about 10 Å (Fig. S2b).
HSE	Haas-Steinberg equation; a partial differential equation (PDE).
HSJE	Haas-Steinberg-Jacob equation; a closed analytical expression (CAE).
I_D	Intensity of the donor fluorescence in the donor-only peptide (eq. S3).
I_{DA}	Intensity of the donor fluorescence in the donor-acceptor peptide (eq. S3).
The *ideal-chain* model	or random-coil model is a distance probability distribution with the single parameter, *b* (eq. 16, eq.18 eq. S27, eq. S28)
J	Augmented diffusion coefficient, dimensionless (eq. 12)
k	Rate constant of fluorescence kinetics that are, in this work, usually monoexponential but, generally, do not have to be. In this work all rate constants are measured in units of 1/ns.
k_D	Rate constant of the donor fluorescence kinetics (not necessarily monoexponential kinetics) after donor excitation and in the absence of an acceptor.

k_{DA}	Rate constant of the donor fluorescence kinetics (not necessarily monoexponential kinetics) after donor excitation and in the presence of an acceptor.
k_{nrad}	Rate constant of donor fluorescence deactivation by external quenching.
k_{rad}	Rate constant of donor fluorescence deactivation by fluorescence emission.
k_{FRET}	Rate constant of donor fluorescence deactivation by FRET (eq. 2, eq. 3).
$k_T\ (r)$	The Förster transfer rate is the rate constant of donor fluorescence deactivation by FRET at donor-acceptor distance, R_{DA} or r (eq. 1).
L	Effective distance in absence of diffusion measured in units of Å (eq. 9).
M	Parameter that describes the steepness of a $DI(X)$ profile (eq. 13).
N	Normalization constant that follows from the condition that the integral of a probability distance distribution from the left integration limit, r_L, to the right integration limit, r_R, has to equal unity or 100% (eqs. 16-19, eq. S26).
$N(r,t)$	Time dependent distance distribution of the optically excited donor and the acceptor in the donor-acceptor peptide after donor excitation.
$N_0(r)$	Normalized initial distance distribution at t = 0.
NAla	Naphtylalanine: This donor usually displays monoexponential fluorescence kinetics after excitation (Fig. S1, Fig. S2a).
NAla sdFRET	A short-distance FRET method that employs the donor-acceptor pair, naphtylalanine and Dbo. The Förster radius, R_0, is only about 10 Å (Fig. S2a).
PDE	Partial differential equation.
$p(r)$	Probability distance distribution measured in units of $Å^{-1}$.
$p_{eq}(r)$	Equilibrium probability distance distribution measured in units of $Å^{-1}$.
r	Donor-acceptor distance in a flexible peptide or molecule measured in units of Å.
r_L	Left integration limit of a distance probability distribution (eqs. 9,10).
r_R	Right integration limit of a distance probability distribution (eqs. 9,10).
R	Effective distance at limitless diffusion (eq. 10).
R_{DA}	Donor-acceptor distance in a molecule with a fixed donor- acceptor distance (eq. 1, eqs. S6a,b)
R_{eff}	Effective donor-acceptor distance (eq. S8, eqs. S9a-b)

R_T	Critical Förster radius; the Förster radius when the donor quantum yield equals unity (eq. 1, eq. S5).
R_0	Förster radius at a specific donor quantum yield.
s	Donor-acceptor distance measured in units of r/R_0, dimensionless (eq. 25).
sdFRET	Short-distance FRET experiments can be based on steady-state as well as time-resolved fluorescence measurements but always use the conjugate (Dbo) of 2,3-diazabicyclo[2.2.2]oct-2-ene (DBO) and asparagine as acceptor. Dbo paired with donors such as NAla, Trp, FTrp or Tyr results in Förster radii in the 10 Å domain.
The "*Skewed-Gaussian*"	model is a 3-D Gaussian probability distance distribution with two parameters (*a* and *b*) (eqs. 16,18).
t	Time after donor excitation measured in units of ns.
X	Square root of the augmented diffusion coefficient J (eq. 12).
X_0	Value of the midpoint of a *DI*(X)-profile; *DI* = 50% at X=X_0 (eq. 13).
z	Time after donor excitation measured in units of t/τ_D, dimensionless (eq. 21).
Φ_D	Donor quantum yield, ranging from 0 to 100% (eqs. S31-33).
τ_D	Donor lifetime in the absence of an acceptor, inverse of $k_{D.}$, measured in units of ns (eqs. 5,6).
τ_{DA}	Donor lifetime in the presence of an acceptor; inverse of k_{DA}, measured in units of ns (comp. Chapter 8a).
τ_{rad}	Radiative donor lifetime: the donor lifetime in the absence of an acceptor or FRET, when the donor quantum yield equals unity; inverse of k_{rad}, (Fig.1, (I), eq. S31).

.

3. How Experiments Lead to the Effective Distance

In Figure S1, we show chemical structures of the donor-acceptor and acceptor-only peptides that we used for “NAla short-distance FRET” (NAla-sdFRET) and for “FTrp short-distance FRET” (FTrp-sdFRET) measurements.[1-4] The corresponding donor fluorescence decay kinetics are shown in Figure S2 and provide k_{DA} and k_D and, by that, k_{FRET} (via equation 2 in the main text: $k_{FRET} = k_{DA}$-k_D). The effective distance is then obtained from equations S1 and S2 (equations 3 in the main text).

$$k_{\text{FRET}} = k_{\text{D}} \left(\frac{R_0}{R_{\text{eff}}} \right)^6 \qquad \text{S1}$$

$$R_{\text{eff}} = R_0 \left(\frac{k_{\text{D}}}{k_{\text{FRET}}} \right)^{1/6} \qquad \text{S2}$$

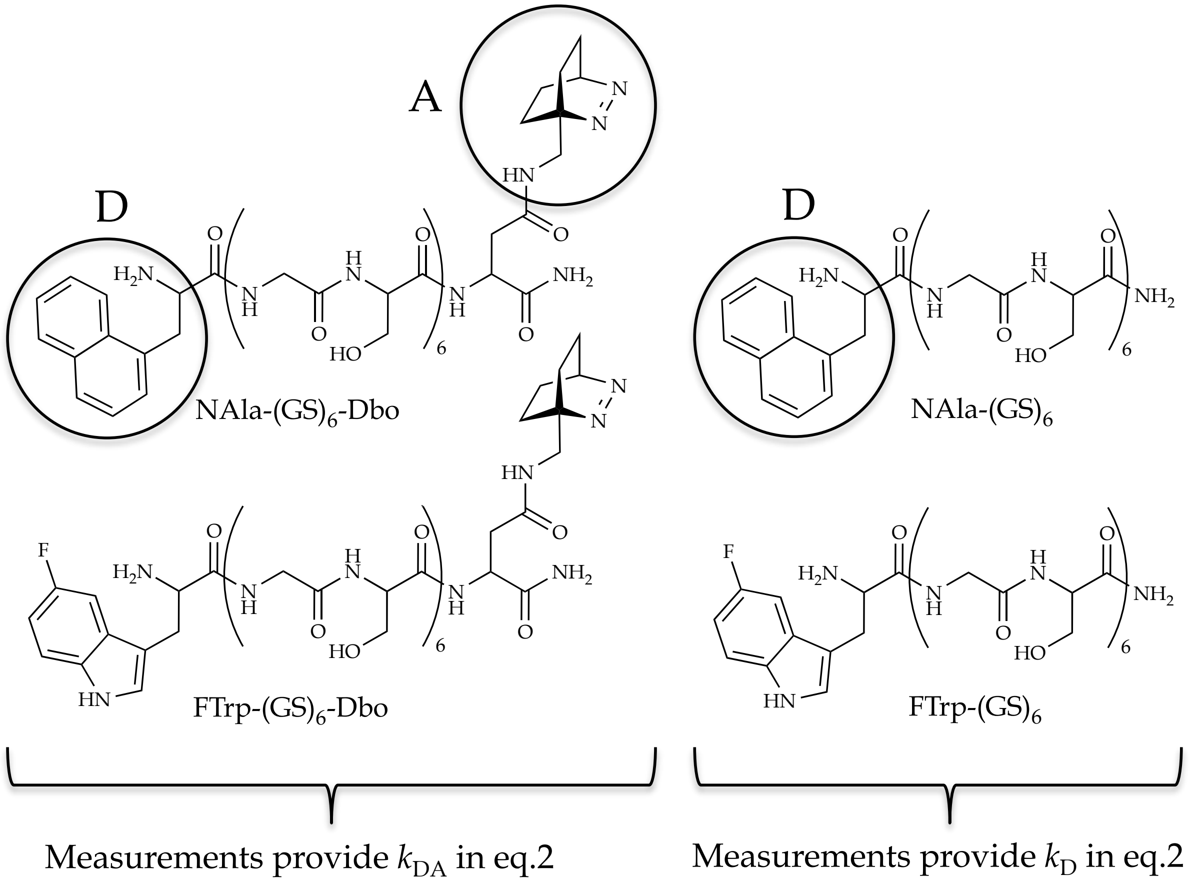

Figure S1. Examplary structures of "donor-acceptor" peptides (left) and "donor-only" peptides (right) used in NAla-sdFRET (top row, the donor, D, and the acceptor, A, are marked by circles) and FTrp-sdFRET (bottom row). The donor-acceptor peptides are used to determine the donor fluorescence lifetime in presence of FRET, k_{DA}. The donor-only peptides are used to determine this lifetime in absence of FRET, k_D. The difference is the FRET rate constant or the effective FRET rate (eq. 2 in the main text).

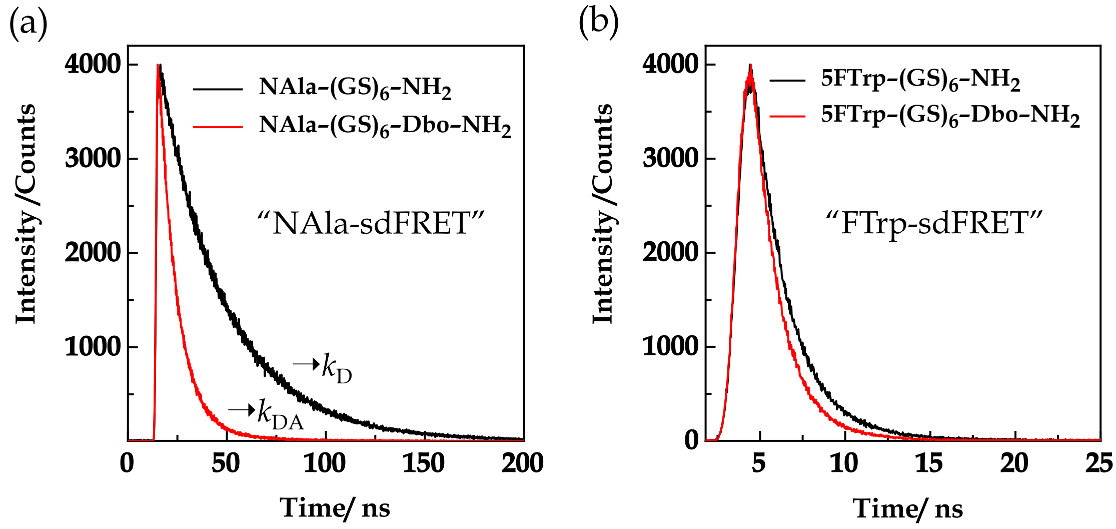

Figure S2. Experimental determination of k_{FRET} and the effective distance by NAla-sdFRET and FTrp-sdFRET. **(a) NAla-sdFRET:** In the absence of acceptor, of FRET, the fluorescence of the donor (Naphtylalanine, NAla) in the donor-only peptide NAla–(GS)$_6$–NH$_2$ decreases after excitation with the rate constant k_D (black trace). In the presence of acceptor, of FRET, the fluorescence of the donor in the donor-acceptor peptide NAla–(GS)$_6$–Dbo–NH$_2$ decreases with the rate constant k_{DA} (red trace). k_{FRET} equals k_{DA} minus k_D (eq. 1). The effective distance is obtained by equations S1 and S2 (eqs. 3 and 4 in the main text). **(b) FTrp-sdFRET**: Same measurements, but with 5-L-Fluorotryptophan (FTrp) as donor.

4. How Theory Leads to the Effective Distance

In other words: "How Solving the HSE Leads to the Effective Distance". In Figure S3 (a-i), we illustrate in detail how the solutions of the HSE look like under a variety of conditions. Our focus is then on the calculation of the effective distance serving as the interface between the theoretical and the experimental results, the data to be fitted. We have shown in the main text's *Introduction* and here *(SI, Chapter 3*) how the effective distance is obtained in experiments and show here *(SI, Chapter 4*) how it is obtained from numerical solutions of the HSE.

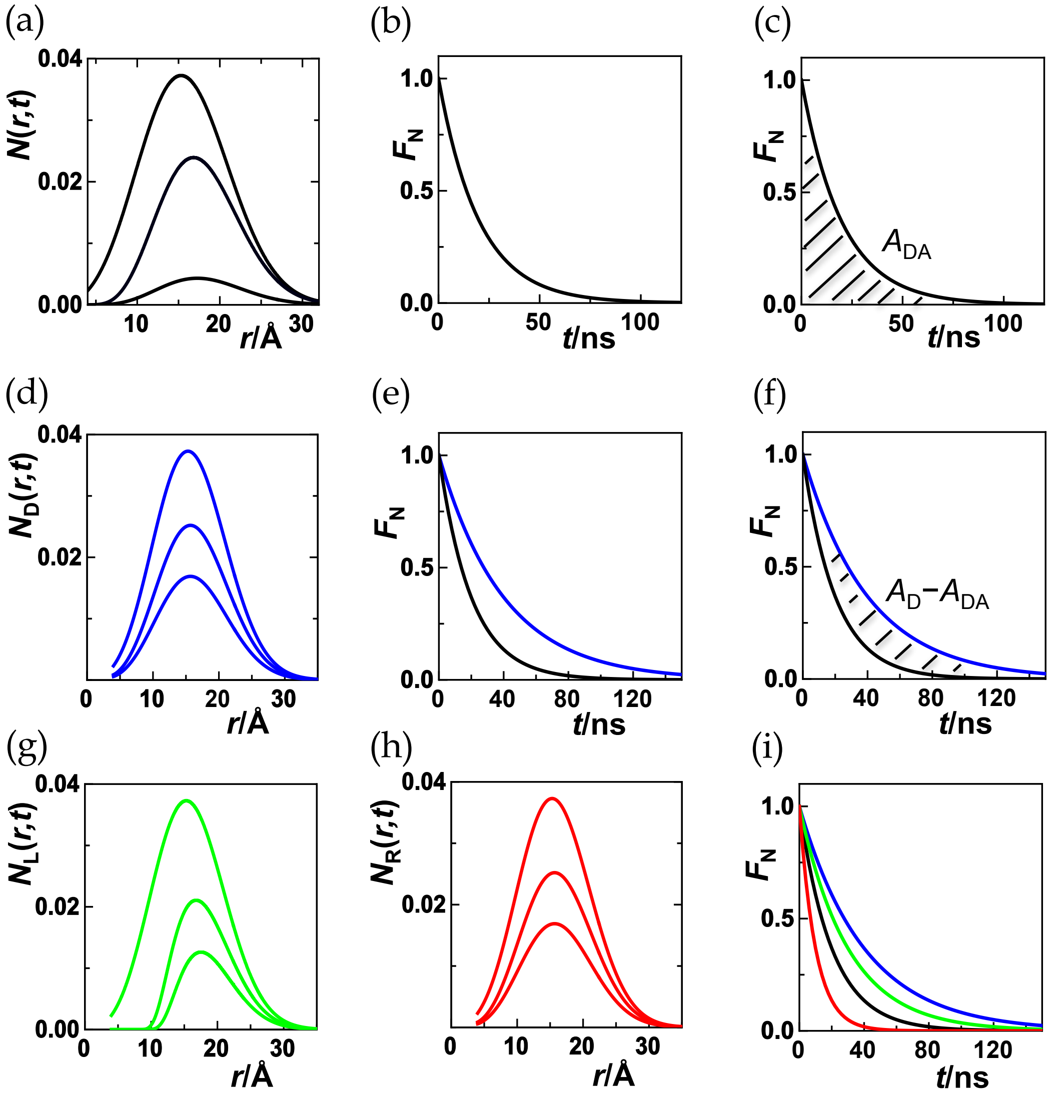

Figure S3. (a) A distance distribution as it develops in time (curves from top to bottom) and in the presence of FRET, of a donor-acceptor pair. **(b)** Time course of the fluorescence intensity normalized to an initial value of 1. **(c)** The area, A_{DA}, under the kinetic curve shown in (b). **(d)** The same distance distribution as it develops in time in the absence of FRET, that is, in the presence of the donor but in the absence of the acceptor. The shape of the distribution remains constant during its decrease. **(e)** Time course of the fluorescence intensity in the presence of donor and in the presence of acceptor (black trace), as well as in the absence of acceptor (blue curve). **(f)** The area A_D minus A_{DA} between the time courses shown in (e). **(g)** The same distance distribution as it develops in time, in the presence of the donor-acceptor pair but in the absence of diffusion. **(h)** The same distance distribution as it develops in time, in the presence of the donor acceptor pair but with extremely high diffusion. The shape of the distribution remains constant during its decrease, as in (d). **(i)** Time courses for four different cases. *Blue trace:* The donor is present, the acceptor absent. *Green trace:* The donor-acceptor pair is present, their mutual diffusion is absent. *Black trace:* The donor-acceptor pair is present, the diffusion coefficient is at an intermediate typical value. *Red trace:* The donor-acceptor pair is present, the diffusion coefficient is extremely high.

Figure S3a shows a solution of the HSE. It provides the time development of a distance distribution in a peptide equipped with a donor and acceptor (subscript: DA). The number of excited chains N^* decreases with time. The distribution maximum shifts slightly to the right as the fluorescence of peptides with shorter donor-acceptor distances is more quickly quenched by FRET as that of peptides with longer donor-acceptor distances, as a consequence of Förster's law (eq. 1 in the main text). In Figure S3b, we follow this development by following the decreasing fluorescence intensity that we obtain by integrating over space, that is, over the donor-acceptor distance, *r*.

Figure S3c is identical to Figure 3b but emphasizes the area under the trace A_{DA}. This area, A_{DA} is proportional to the emitted steady-state fluorescence. When we do the same experiments with the donor-only peptide (Fig. S3d), we have no FRET as the acceptor is absent. Because of that, the statistical-weight distance distribution shrinks but retains its shape, the normalized probability distance distribution (not shown) does not change at all. There is no shift of the maximum to the right. The rate constant of deactivation is, of course, slower in the absence of FRET (blue curve) than in its presence (black curve, Fig. S3e.) The area under this kinetic trace A_D is therefore larger. The difference of the areas is $A_D - A_{DA}$ (Fig. S3f) and presents a measure of the fractional fluorescence intensity quenched by FRET. Figure S3g illustrates the HSE solutions for a donor-acceptor peptide in the presence of FRET and in the absence of diffusion. We observe a considerable shift of the maximum to the right. Figure S3h shows the same but in the presence of infinite diffusion. Peptides with short donor-acceptor distance are deactivated first but are instantly replenished. Such strong diffusion maintains the shape of the distance distribution. Figure S3i illustrates how fast the fluorescence decreases in each case: fastest at infinite diffusion (red), second fastest at intermediate diffusion (black), slow without diffusion (green), and slowest in the absence of FRET (blue).

A central parameter in any FRET analysis is usually the energy transfer efficiency which is the answer to the question of how many donor deexcitation events are caused by FRET in comparison to all donor-deactivation events. It is usually given by equation S3 which compares the fluorescence intensities of the donor-acceptor and donor-only peptides in steady-state experiments. We also investigate this basic concept of the energy transfer

efficiency in the *Supporting Information, Chapter 5*. As the emitted fluorescence is, as stated, proportional to the area under the fluorescence decay curve, we can also write equation S4. Thus, the numerical solution of the HSE provides us with the predicted energy transfer efficiency.

In a situation, where the distance of donor and acceptor doesn't change in time, the transfer efficiency is also given by equation S5. In such a case Förster's law is valid (eq. 1) and we obtain the donor-acceptor distance, provided we know R_0, by equations S6a-b. In a situation where donor and acceptor can change their distance during the lifetime of the donor fluorescence, we simply use the analogous equations S7-9.[1] Thus, if we know the fluorescence intensity from steady-state experiments or if we know the fluorescence kinetics from lifetime experiments we get the experimentally observed effective distance by equation 9b. In case we know A_{DA} and A_D by numerical solutions of the HSE we get the predicted or calculated effective distance from equations S4 and S9b.

$$E = \frac{I_D - I_{DA}}{I_D} \tag{S3}$$

$$E = \frac{A_D - A_{DA}}{A_D} \tag{S4}$$

$$E = \frac{k_T}{k_T + k_D} \tag{S5}$$

$$k_T = k_D \left(\frac{R_0}{R_{DA}} \right)^6 \tag{1}$$

$$E = \frac{R_0^6}{R_0^6 + R_{DA}^6} \tag{S6a}$$

$$R_{DA} = R_0 \left(\frac{1}{E} - 1 \right)^{1/6} \tag{S6b}$$

$$E = \frac{k_{FRET}}{k_{FRET} + k_D} \tag{S7}$$

$$k_{\mathrm{FRET}} = k_{\mathrm{D}} \left(\frac{R_0}{R_{\mathrm{eff}}} \right)^6 \quad \text{(S8)}$$

$$E = \frac{R_0^6}{R_0^6 + R_{\mathrm{eff}}^6} \quad \text{(S9a)}$$

$$R_{eff} = R_0 \left(\frac{1}{E} - 1 \right)^{1/6} \quad \text{(S9b)}$$

5. Fundamental Concepts

5a. The Concept of the Energy Transfer Efficiency

To prepare the derivations of the *L*- and *R*-equations (equations 8 and 9 in the main text), we shortly recall two basic concepts, the concept of the energy transfer efficiency and the concept of the average.

A central observable in every FRET study is the energy transfer efficiency, *E*, the number of donor deactivation events caused by FRET divided by all donor deactivation events. Experimentally, it can be determined in steady-state experiments by measuring the fluorescence intensities, I_{D} and I_{DA}, of the donor in absence and in presence of the acceptor, that is, in the donor-only or D-peptide and in the donor-acceptor or DA-peptide. The intensity fraction quenched by FRET is then given by equation S10.,

$$E = \frac{I_{\mathrm{D}} - I_{\mathrm{DA}}}{I_{\mathrm{D}}} \quad \text{(S10)}$$

In time resolved experiments, the initial amplitude at t=0 of a decay kinetic can be set to unity. The areas, A_{D} and A_{DA} under the kinetic traces of the donor-only and donor-acceptor peptide are then proportional to the emitted fluorescence, and the fraction of fluorescence quenched by FRET is given by equation S11.

$$E = \frac{A_{\mathrm{D}} - A_{\mathrm{DA}}}{A_{\mathrm{D}}} \quad \text{(S11)}$$

This experimentally determined transfer efficiency serves to define k_{FRET} (eqs. S12a,b) and the effective distance (eqs. S9a,b). Thus, k_{FRET} should not be mistaken for a rate constant that can only describe a purely monoexponential decay process.

$$E = \frac{k_{\text{FRET}}}{k_{\text{FRET}} + k_{\text{D}}} \rightarrow k_{\text{FRET}} = k_{\text{D}} \frac{E}{1-E} \qquad \text{(S12a,b)}$$

$$E = \frac{R_0^6}{R_0^6 + R_{\text{eff}}^6} \rightarrow R_{\text{eff}} = R_0 \left(\frac{1}{E} - 1 \right)^{1/6} \qquad \text{(S9a,b)}$$

Equation S9b will be our starting point to derive the analytical expression for L (Chapter 6a). We just need an expression for E that we can insert.

When we eliminate E in equations S9a and S12a, we obtain equation S13a (eq. 3 in the main text), which we can again solve for the effective distance (eq. S13b, eq. 4 in the main text), in perfect analogy to Förster's law (eq. 1 in the main text.)

$$k_{\text{FRET}} = k_{\text{D}} \left(\frac{R_0}{R_{\text{eff}}} \right)^6 \rightarrow R_{\text{eff}} = R_0 \left(\frac{k_{\text{D}}}{k_{\text{FRET}}} \right)^{1/6} \qquad \text{(S13a,b)}$$

Equation S13b will be our starting point to derive the analytical expression for R (Chapter 6b).

5b. The Concept of the Average

The second concept is the average and its definition is essential in both derivations. The discrete case is usually familiar: When we throw a dice, the numbers N=1,2,…,6 can appear, each with a probability of 1/6, that is, $p(i{=}1){=}p(\text{case 1}){=}1/6$, $p(i{=}2){=}p\ (\text{case 2})=1/6$ and so forth. It is a characteristic of a perfect dice that the probability of each possible outcome or case is the same and is 1/6 but usually $p(i)$ varies. The average in this simple example is obviously 3.5 but in more complicated cases a generally valid formalism should be applied:

$$\langle N \rangle = \sum_{i=1}^{i=6} \left(N_i \cdot p(i) \right) = \sum_{i=1}^{i=6} \left(N \cdot 1/6 \right) = 1/6 \sum_{i=1}^{i=6} N = 1/6 \left(1+2+\ldots+6 \right) = 3.5$$

In the continuous case, the summation over all possible outcomes has to be replaced by integration: If we have a dependent variable A, which changes continuously with the variable x as described by the probability density distribution $p(x)$, the average of A(x) equals:

$$\langle A(x) \rangle = \int_{x_{\text{L}}}^{x_{\text{R}}} A(x) p(x) \text{dx} \qquad \text{(S14)}$$

We will use this definition to obtain the average energy transfer efficiency in the *L*-derivation and to obtain the FRET rate, k_{FRET}, as the distance average of the Förster transfer rate, k_{T}, in the *R*-derivation.

6. Extreme Effective Distances

6a. The Effective Distance, *L*, in Absence of Diffusion

The effective distance, *L*, in absence of diffusion is a function of the Förster radius and the distance distribution, $L=f(R_0,p(r))$ (eq. S16). We derived this equation in the *Supporting Information* of reference [2]. (eq. S15, eq. 9 in the main text).

$$L = R_{\mathrm{eff},\,D\to 0} = R_0 \left(\left(1 - \int_{r_{\mathrm{L}}}^{r_{\mathrm{R}}} p(r) \left(\frac{r^6}{r^6 + R_0^6} \right) \mathrm{d}r \right)^{-1} - 1 \right)^{1/6} \quad \text{(S15)}$$

If we have a distance distribution $p(r)$ and no diffusion and if we look only at a specific distance *r*, we have equation S16 (identical to equation S6a as $r = R_{\mathrm{DA}}$).

$$E(r) = \frac{R_0^6}{R_0^6 + r^6} \quad \text{(S16)}$$

Thus, we obtain values close to unity at very short distances, $r \to 0$, and values close to zero at large distances, $r \to \infty$. As the observed energy transfer efficiency accounts for the deactivation events at all distances it is equal to the distance average of the distance-dependent energy transfer efficiency (eq. S17).

$$E = \langle E(r) \rangle = \int_{r_{\mathrm{L}}}^{r_{\mathrm{R}}} E(r) p(r) \mathrm{d}r \quad \text{(S17)}$$

We insert equation S16 and obtain

$$E = \langle E(r) \rangle = \int_{r_{\mathrm{L}}}^{r_{\mathrm{R}}} \frac{R_0^6}{R_0^6 + r^6} p(r) \mathrm{d}r = \int_{r_{\mathrm{L}}}^{r_{\mathrm{R}}} \left(1 - \frac{r^6}{R_0^6 + r^6} \right) p(r) \mathrm{d}r = 1 - \int_{r_{\mathrm{L}}}^{r_{\mathrm{R}}} \left(\frac{r^6}{R_0^6 + r^6} \right) p(r) \mathrm{d}r$$

where we used in the second to last step that a constant does not depend on any variable; thus, the average of the constant taken over any variable can only be the constant itself. For instance: $\int_{r_L}^{r_R} 1 \cdot p(r) \mathrm{d}r = 1$

Thus, we now have:

$$E = 1 - \int_{r_L}^{r_R} \left(\frac{r^6}{R_0^6 + r^6} \right) p(r) \mathrm{d}r \tag{S18}$$

Finally, we insert equation S18 into equation S9b and obtain the expression for L (eq. S19, repeated eq. 15, eq. 9 in the main text). This derivation has firstly been published in the Supporting Information of reference S1 (ref. 12 in the main text) .

$$L = R_{\mathrm{eff},\, D \to 0} = R_0 \left(\left(1 - \int_{r_L}^{r_R} p(r) \left(\frac{r^6}{r^6 + R_0^6} \right) \mathrm{d}r \right)^{-1} - 1 \right)^{1/6} \tag{S19}$$

6b. The Effective Distance, *R*, at Infinite Diffusion

The effective distance at infinite diffusion, R, is a function of only the distance distribution, $R=f(p(r))$, (eq. S20, eq. 10 in the main text). In contrast to L, the function R is independent of the donor quantum yield and the Förster radius. Thus, with growing diffusion, the effective distance depends more and more only on the distance distribution.

$$R = R_{\mathrm{eff},\, D \to \infty} = \left\langle r^{-6} \right\rangle^{-1/6} = \left(\int_{r_L}^{r_R} \left(1 / r^6 \right) p(r) \mathrm{d}r \right)^{-1/6} \tag{S20}$$

What characterizes the case of infinite diffusion is that the distance dependence of Förster's law, that is, the distance-dependent intensity decay caused by FRET, is not able to distort the shape of the distance distribution. Due to limitless diffusion, the probability distribution of donor-acceptor distances becomes time-independent and remains unchanged. This is only possible if the same rate constant, k_{DA}, is effective at every distance. Part of k_{DA} is k_{D}, the rate constant in absence of FRET, which is in any case time-independent. The other part is k_{FRET} because $k_{\mathrm{DA}}=k_{\mathrm{D}}+k_{\mathrm{FRET}}$. Peptides with donor-acceptor distances at distance r become deactivated as governed by the rate constant $k_{\mathrm{D}}+\mathrm{k_T}(r)$. However, in the case of limitless diffusion, the distance, at which any peptide molecule is deactivated by FRET, is only controlled by the distance distribution, $p(r)$, and is otherwise completely random. Thus, the

FRET rate constant connected to the deactivation of any molecule is also only controlled by the distance distribution, $p(r)$; and k_{FRET} is therefore equal to k_T averaged over all possible distances as weighted by the distance distribution (eq. S21).

$$k_{\mathrm{FRET}} = \langle k_{\mathrm{T}} \rangle = \int_{r_{\mathrm{L}}}^{r_{\mathrm{R}}} k_{\mathrm{T}}(r) p(r) \mathrm{d}r \tag{S21}$$

When we insert Förster's law, we obtain equation S22.

$$k_{\mathrm{FRET}} = \int_{r_{\mathrm{L}}}^{r_{\mathrm{R}}} k_{\mathrm{D}} \frac{R_0^6}{r^6} p(r) \mathrm{d}r = k_{\mathrm{D}} R_0^6 \int_{r_{\mathrm{L}}}^{r_{\mathrm{R}}} \frac{1}{r^6} p(r) \mathrm{d}r = k_{\mathrm{D}} R_0^6 \langle r^{-6} \rangle \tag{S22}$$

Finally, we start with equation S5b for the effective distance, apply equation S22, and obtain the expression for R (eq. S23 , repeated eq. 20, eq. 10 in the main text)

$$R_{\mathrm{eff}} = R_0 \left(\frac{k_{\mathrm{D}}}{k_{\mathrm{FRET}}} \right)^{1/6} = R_0 \left(\frac{k_{\mathrm{D}}}{k_{\mathrm{D}} R_0^6 \langle r^{-6} \rangle} \right)^{1/6} = \langle r^{-6} \rangle^{-1/6} \text{ , or:}$$

$$R = R_{\mathrm{eff},\, D\to\infty} = \langle r^{-6} \rangle^{-1/6} = \left(\int_{r_{\mathrm{L}}}^{r_{\mathrm{R}}} \left(1/r^6 \right) p(r) \mathrm{d}r \right)^{-1/6} \tag{S23}$$

With both values analytically determined, we now have a very natural method at hand to calculate diffusion-influence or DI values ranging from 0 to 100% via equation S24 (equation 10 in the main text.)

$$DI = \frac{R_{\mathrm{eff}} - L}{R - L} \tag{S24}$$

7. Explanatory Notes On the Equivalence Statements

Before we add explanatory notes to the equivalence statements we start with a survey.

7a. Survey of the Equivalence Statements

Next to knowing the extreme values of the effective distance, the equivalence statements were decisive in developing the HSJE. Enforced by logic, the first three statements yield the fourth. First, we repeat the formal expressions of these four statements. Later, we add explanatory notes.

We arrived at the equivalence statements by always comparing two diffusion-impact profiles obtained with two different sets of HSE-inputs, where each set contains the probability distance distribution, the diffusion coefficient, the Förster radius and the left integration limit. We repeat that we never noted an influence of the right-integration limit, r_R. We just had to make sure that $p(r_R)$ is always close to zero. Thus, set[(1)] consists of $p^{(1)}(r)$, $D^{(1)}$, $\tau_D^{(1)}$, $R_0^{(1)}$ and $r_L^{(1)}$ and set[(2)] of $p^{(2)}(r)$, $D^{(2)}$, $\tau_D^{(2)}$, $R_0^{(2)}$ and $r_L^{(2)}$. The augmented diffusion coefficients, $J^{(1)}$ and $J^{(2)}$, are then given by equation S25 (in accordance with equation 12 in the main text).

$$J^{(1)} = \frac{D^{(1)}\tau_D^{(1)}}{\left(R_0^{(1)}\right)^2} \text{ and } J^{(2)} = \frac{D^{(2)}\tau_D^{(2)}}{\left(R_0^{(2)}\right)^2} \tag{S25}$$

The survey:

ES1: *The diffusion-impact profiles of any two well-behaved probability distributions,* $p^{(1)}(r)$ and $p^{(2)}(r)$, *coincide if they were obtained with the same values of the donor lifetime,* τ_D, *the Förster radius,* R_0, *and the left-integration limit,* r_L.

$$\text{For any } p^{(1)}(r) \text{ and } p^{(2)}(r): \text{If } D^{(1)} = D^{(2)},\ \tau_D^{(1)} = \tau_D^{(2)},\ R_0^{(1)} = R_0^{(2)}, \text{and } r_L^{(1)} = r_L^{(2)} \text{ then } DI^{(1)}\left(J^{(1)}\right) = DI^{(2)}\left(J^{(2)}\right) \tag{ES1}$$

ES2: *Given any probability distribution: If the diffusion coefficient is varied from* $D^{(1)}$ *to* $D^{(2)}$ *and the donor lifetime from* $\tau_D^{(1)}$ to $\tau_D^{(2)}$ *but the product of diffusion coefficient and donor lifetime remains constant, then the diffusion-impact values obtained from the diffusion-impact functions,* $DI^{(1)}(J^{(1)})$ *and* $DI^{(2)}(J^{(2)})$, *are identical. The corresponding diffusion-impact profiles coincide.*

$$\text{For any } p^{(1)}(r) = p^{(2)}(r): \text{If } D^{(1)}\tau_D^{(1)} = D^{(2)}\tau_D^{(2)},\ R_0^{(1)} = R_0^{(2)}, \text{ and } r_L^{(1)} = r_L^{(2)} \text{ then } DI^{(1)}\left(J^{(1)}\right) = DI^{(2)}\left(J^{(2)}\right) \tag{ES2}$$

ES3: *Given any probability distribution: If the Förster radius and the left-integration limit are varied but not their ratio then the diffusion-impact values obtained from the diffusion-impact functions,* $DI^{(1)}(J^{(1)})$ *and* $DI^{(2)}(J^{(2)})$, *are identical. The corresponding diffusion-impact profiles coincide.*

$$\text{For any } p^{(1)}(r) = p^{(2)}(r): \text{ If } \frac{D^{(1)}\tau_D^{(1)}}{\left(R_0^{(1)}\right)^2} = \frac{D^{(2)}\tau_D^{(2)}}{\left(R_0^{(2)}\right)^2} \text{ and } \frac{R_0^{(1)}}{r_L^{(1)}} = \frac{R_0^{(2)}}{r_L^{(2)}} \text{ then } DI^{(1)}\left(J^{(1)}\right) = DI^{(2)}\left(J^{(2)}\right) \tag{ES3}$$

ES4: *For any pair of distance distributions,* $p^{(1)}(r)$ and $p^{(2)}(r)$: *If the diffusion coefficient or the donor lifetime or the Förster radius are varied but not the augmented diffusion coefficient J composed of these parameters and if the ratio of Förster radius and left-integration limit is not varied, then the diffusion-impact values obtained from the diffusion-impact functions,* $DI^{(1)}(J^{(1)})$ *and* $DI^{(2)}(J^{(2)})$*, are identical. The corresponding diffusion-impact profiles coincide.*

For any $p^{(1)}(r)$ and $p^{(2)}(r)$: If $\frac{D^{(1)}\tau_{\mathrm{D}}^{(1)}}{\left(R_0^{(1)}\right)^2}=\frac{D^{(2)}\tau_{\mathrm{D}}^{(2)}}{\left(R_0^{(2)}\right)^2}$ and $\frac{R_0^{(1)}}{r_{\mathrm{L}}^{(1)}}=\frac{R_0^{(2)}}{r_{\mathrm{L}}^{(2)}}$ (ES4)

then $DI^{(1)}\left(J^{(1)}\right)=DI^{(2)}\left(J^{(2)}\right)$

7b. ES1: The Impact of the Left Integration Limit, r_{L}

We repeat the formal notation of ES1:

For any $p^{(1)}(r)$ and $p^{(2)}(r)$: If $D^{(1)}=D^{(2)}$, $\tau_{\mathrm{D}}^{(1)}=\tau_{\mathrm{D}}^{(2)}$, $R_0^{(1)}=R_0^{(2)}$, and $r_{\mathrm{L}}^{(1)}=r_{\mathrm{L}}^{(2)}$

then $DI^{(1)}\left(J^{(1)}\right)=DI^{(2)}\left(J^{(2)}\right)$

The *DI*-profiles of two distributions overlap, but only, if they share the same left integration limit, r_{L}. In Figure S1, we illustrate what happens if everything but the left integration limit stays the same. We start with two distributions (Fig. S1a), whose r_{L}-values differ. Figure S1b shows the corresponding diffusion profiles and Figure S1c the diffusion-impact profiles. These profiles do not overlap. We observe that the distribution with the smaller r_{L} value approaches the dynamic limit more slowly.

The contribution of diffusion to FRET at infinite diffusion is higher in case of the smaller left integration value. This can be concluded from term-(2) of the HSE, which encompasses Förster's law (eq. 5): At small r_{L} values, FRET becomes fast and, therefore, higher values of the augmented diffusion coefficient are necessary to reach the dynamic limit.

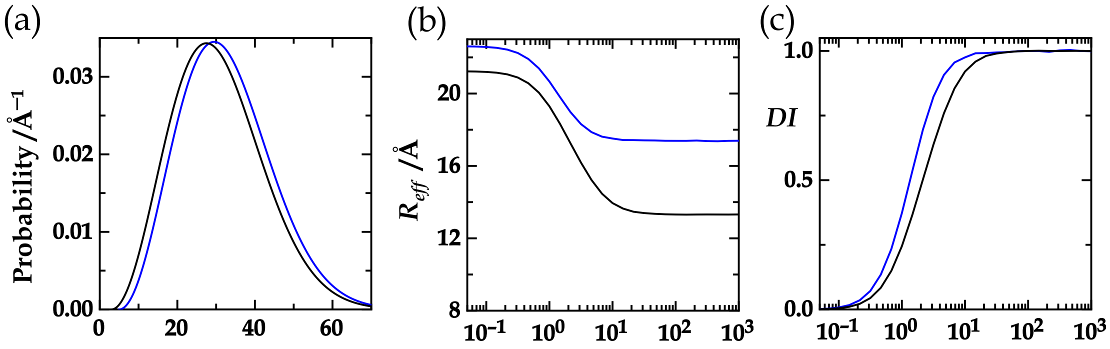

Figure S4. (a) Two Gaussian distance distributions (see equation 18 in the main text) with b = 30Å, r_L = 3Å (black) and b = 30 Å, r_L = 5Å (blue). (**b**) The corresponding diffusion profiles obtained with τ_D = 100ns and R_0 = 15Å. **(c)** The corresponding *DI*-profiles.

7c. ES1: "Well-Behaved Distance Distributions"

Such that a distance distribution is really a probability density distance distribution, its integral from start ($r = r_L$) to end ($r = r_R$) has to be unity (eq. S26).

$$\int_{r_L}^{r_R} p(r)\,dr = 1 \qquad \text{(S26)}$$

The left-integration limit can usually not be chosen as zero, because donor and acceptor can impossibly occupy the same point in space. In the past, the r_L value has therefore been chosen somewhat arbitrarily or has not been mentioned at all. If we now use, for instance, the ideal-chain model as expressed by equation S27, we obtain in some case large discontinuities of the distance probability density at the point $r = r_L$ (Fig. S2a); it would be hard to find any physical justification for such a discontinuity. If instead we use the distribution model as expressed by equation S28, the probability density value starts with zero at r=r_L and continuously grows or descends thereafter (Fig. S2b).

$$p(r) = Nr^2 \cdot \left(\frac{3}{2\pi b^2}\right) \cdot \exp\left(-\frac{3r^2}{2b^2}\right);\ b = \left\langle r^2 \right\rangle^{1/2} \qquad \text{(S27)}$$

$$p(r) = Nr_0^2 \cdot \left(\frac{3}{2\pi b^2}\right) \cdot \exp\left(-\frac{3r_0^2}{2b^2}\right);\ r_0 = r - r_L \qquad \text{(S28)}$$

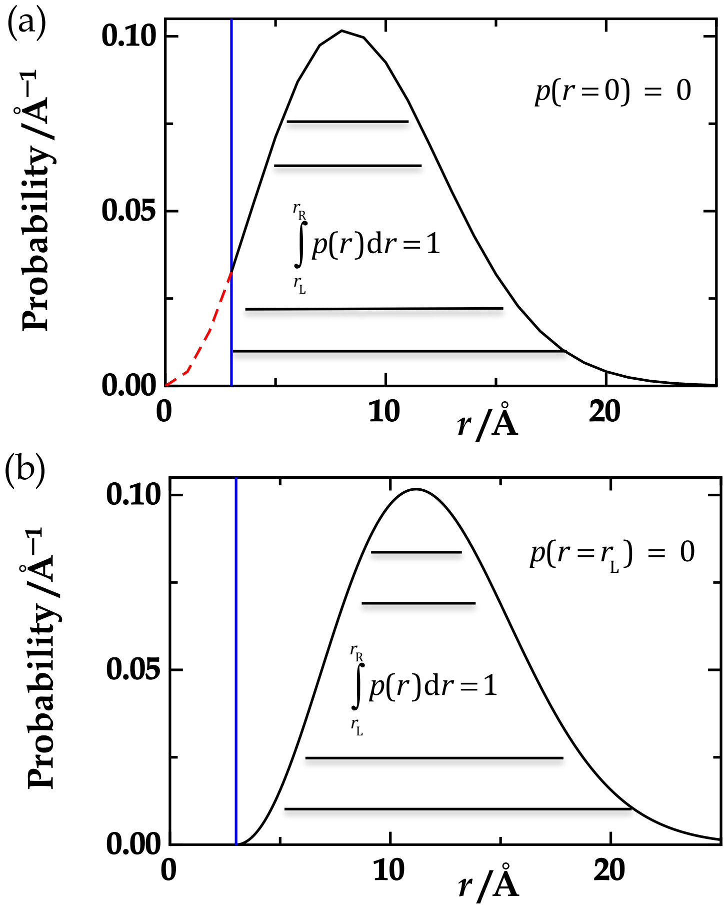

Figure S5. (a) A distance distribution according to equation S27 with b=10Å. The probability density equals zero at r=0Å, but the smallest donor-acceptor distance allowed is r_L = 3Å. The normalization is taken from r_L to r_R such that the area under the curve between r_L and r_R becomes unity. The probability density jumps from zero when $r < r_L$ to a large finite value at $r = r_L$. **(b)** A distance distribution according to equation S28 with b = 10Å. The probability density equals zero at $r = r_L$. After normalization, the area under the curve between r_L and r_R equals unity. The probability density value starts with zero at $r = r_L$ and continuously grows and descends thereafter.

7d. ES2: Variation of the Donor Lifetime

For any $p^{(1)}(r) = p^{(2)}(r)$: If $D^{(1)}\tau_D^{(1)} = D^{(2)}\tau_D^{(2)}$, $R_0^{(1)} = R_0^{(2)}$, and $r_L^{(1)} = r_L^{(2)}$
then $DI^{(1)}\left(J^{(1)}\right) = DI^{(2)}\left(J^{(2)}\right)$

ES2 follows by force from the HSE, as shown in the main text by variable substitution. We can also convince ourselves of ES2 in more intuitive ways. The simplest procedure is probably to simply multiply both sites of the HSE with the same factor, c, and absorb this factor on the right hand site into the donor lifetime in term-(1) and also in term-(2) and into

the diffusion coefficient in term-(3). We obtain a new donor lifetime τ_D' and a new diffusion coefficient, D', but their product has the same value as the product of the old donor lifetime and the old diffusion coefficient because: $\tau_D' \cdot D' = (\tau_D/c) \cdot (D \cdot c) = \tau_D \cdot D$. By multiplying both sites with, for instance, $c = 10$, we just make all processes in the HSE ten times faster. The effective-distance values we obtain from the HSE would, of course, not be impacted by that procedure. If we wish to take it to a more universal or or nearly "poetic" level: If all processes in the universe would suddenly run ten times faster, we would not be able to notice this change, as all our brain processes would also run ten times faster.

7e. The HSJE Written as a Single Equation

In Figure 8 in the main text we represented the HSJE as decomposed into eight simple equations (I –VIII). That such a decomposition makes sense becomes obvious when we try here to represent the equation on a single line (eq. S29).

(S29)

$$R_{\text{eff}} = \frac{\left(\dfrac{\sqrt{\dfrac{D\tau_D}{R_0^2}}}{a_0 + a_1\left(\dfrac{R_0}{r_L}\right) + a_2\left(\dfrac{R_0}{r_L}\right)^2}\right)^{\left(b_0 + b_1\left(\frac{R_0}{r_L}\right) + b_2\left(\frac{R_0}{r_L}\right)^2\right)}}{1 + \left(\dfrac{\sqrt{\dfrac{D\tau_D}{R_0^2}}}{a_0 + a_1\left(\dfrac{R_0}{r_L}\right) + a_2\left(\dfrac{R_0}{r_L}\right)^2}\right)^{\left(b_0 + b_1\left(\frac{R_0}{r_L}\right) + b_2\left(\frac{R_0}{r_L}\right)^2\right)}} \cdot \left(\left(\int_{r_L}^{r_R} \left(1/r^6\right) p(r)\,\mathrm{d}r \right)^{-1/6} - R_0 \left(\left(1 - \int_{r_L}^{r_R} p(r) \left(\frac{r^6}{r^6 + R_0^6} \right) \mathrm{d}r \right)^{-1} - 1 \right)^{1/6} \right) + R_0 \left(\left(1 - \int_{r_L}^{r_R} p(r) \left(\frac{r^6}{r^6 + R_0^6} \right) \mathrm{d}r \right)^{-1} - 1 \right)^{1/6}$$

8. Global Analysis

8a. A Study with NAla sdFRET and FTrp sdFRET on a Flexible Peptide

The following experiments were already reported in detail in reference [3]. We collected 48 data sets on the Donor−GS_6−Dbo peptide where the donor was either FTrp in FTrp sdFRET measurements or NAla in NAla sdFRET measurements (Fig.S1). While FTrp displays a radiative donor lifetime of 19.8 ns in water, NAla displays one of 256 ns. Thus, we switched

the donor-acceptor pair from FTrp/Dbo to NAla/Dbo and varied, by that, the donor fluorescence lifetime by a factor of about 13, by more than an order of magnitude. Also by an order of magnitude, by a factor of about 14, we varied the viscosity, η, by adding ethylene glycol to the peptide samples. By that, we varied the diffusion coefficient in accordance with the equation $D=D_0\cdot\eta_0/\eta$, where D_0 and η_0 are the diffusion coefficient and the viscosity in water in the absence of ethylene glycol. Thus, in total we varied the diffusion-enhancement of FRET, varied DFRET, by more than two orders of magnitude.

Each of the so obtained 48 data sets consisted of four values, of the donor lifetime, the Förster radius, the viscosity and the effective distance. At all ethylene glycol concentrations, the kinetic traces followed monoexponential time courses. Note, that we used 5-L-fluorotryptophan (FTrp) instead of tryptophan as donor, as tryptophan would have led to more complex kinetics [5]. Only at high ethylene glycol concentrations and in the case of the FTrp-labeled peptide, biphasic fits seemed to be slightly more appropriate and were used to calculate amplitude-weighted lifetimes, $\tau_{DA} = \sum_i A_i \tau_I$ [6-8]. The rate constants, k_D and k_{DA} were then simply taken as the reciprocal values of the determined lifetime constants, $k_D = \tau_D^{-1}$ and $k_{DA} = \tau_{DA}^{-1}$.

These experimentally obtained effective distance values were now fitted to the theoretical values provided by the HSJE by allowing the software to adjust the three parameters, b, r_L, and D, when the distribution model was the ideal chain model or to adjust the four parameters, a, b, r_L, and D, when the distribution model was the skewed Gaussian model.

8b. Details of the Global Analysis Procedure

Being familiar with global analyses is not a matter of course. Some motivated readers might find the following description useful. In our concrete case, we have 48 experimental data sets, where each set contains the values of the effective-distance, $R_{eff,obs}$, the donor lifetime, $\boldsymbol{\tau}_D$, the Förster radius, R_0, and the viscosity, η. To extract from these data the diffusion coefficient, D, and the parameters of the chosen distance-distribution model, requires a global analysis that can be carried out in Excel and Excel's solver add-in. Thus, we start in Excel with four columns, with the "$R_{eff,obs}$"-, the "$\boldsymbol{\tau}_D$" -, the "R_0"-, and the "η"-column each with 48 entries, thus, each stretched over 48 rows.

When we use the skewed-Gaussian distance distribution model with its two parameters, *a* and *b*, we want to determine the values for *a, b,* r_L and *D*. Of course, as in any fitting, we can also fix one of these parameters, for instance, the left-integration limit, r_L, if we have beforehand information from other sources. As in any fit, we have to choose initial values for the four parameters that we enter into these four "destination" cells, the "*a*"-, "*b*"-, "r_L"-, and "*D*"-cell. The solver varies these fitting parameters in trying to minimize the value of the sum-of square difference between the measured values of the effective distance, $R_{eff,obs}$, and the predicted values, $R_{eff,calc}$, calculated from the HSJE. Let's recall that we explained in the main text's *Introduction* how R_{eff} or $R_{eff,obs}$ values are obtained experimentally (supported by Fig. S1) and in *the Supporting Information, Chapter 4* how R_{eff} or $R_{eff,calc}$ values are obtained from the HSE.

Figure 8 in the main text and equation S29 show how $R_{eff,calc}$ is obtained from the HSJE. To calculate $R_{eff,calc}$ for each of the 48 measured data sets, we need the *L* and *R* values and the *DI* values for each measurement (Fig. 8, (I)). The initially chosen probability-distribution values, *a*, *b*, and r_L together with the experimentally known R_0–values, with the "R_0" –column, give us the *L* and *R*-values for each measurement, the "*L*" and "*R*"-columns (Fig.8, (VIII) and (IX)). The needed integrations to calculate *L* and *R* are done numerically by going in steps of 0.1Å from $r = r_L$ to $r = 60$ Å.

The R_0 values together with the initially chosen r_L value give us the X_0 and *M* values (Fig.8, VI and VII), which then yield the *DI* values or the "*DI*"-column (Fig.8, (II)).

We also need the *X* values for *DI*-calculation, which we obtain from equations III and IV in Figure 8. The diffusion values in equation IV, $D_{1\text{-}48}$, are given through equation S30 by the known viscosity values, by the "η"-column, and by the diffusion value, D_1, which is the diffusion coefficient in pure water, whose initially estimated value is entered into the "*D*"-or "D_1" destination cell before the fit starts and alters it.

$$D_{1-48} = D_1 \cdot \frac{\eta_1}{\eta_{1-48}} \qquad \text{(S30)}$$

The values of the donor lifetime and R_0 are also experimentally known, the "τ_D" –column and the "R_0"-column. So, we have the *J*-and *X*-values or columns, and, by that, the *DI* values or

the "DI"-column and, by that, the calculated effective distance values or the "$R_{eff,calc}$"-column. While the four measurement columns ($R_{eff,obs}$, τ_D, R_0, η) never change during the global fit, the columns containing the derived parameters change whenever the solver changes a value in one of the four destination cells that contain the parameters to be determined, usually a, b, D, and r_L. The solver stops when the value in the target cell can not be further minimized. This value is the sum-of square difference between the measured values of the effective distance, $R_{eff,obs}$, and the predicted values, $R_{eff,calc}$, calculated from the HSJE. In case of a near perfect fit, this value approaches zero.

8c. Applying the HSJE to Experimental Data in a Global Fit

We are ready to present the analysis of this first application of the HSJE. For the peptides shown in Figure S1, we used NAla and FTrp sdFRET to determine 48 data quadruples of the parameters R_{eff}, τ_D, R_0 and η, the viscosity. These data have been formerly presented and discussed in reference [3]. But only now we can apply the HSJE to them. Firstly, we performed the global fit on the basis of the one-parameter ideal chain model (eq. 18 in the main text); the results were always obtained in less than 15 seconds. The fit as shown and described in Figure S6, in panels b and c and the legend, was agreeable but far from perfect. Secondly, we used the 3D- or skewed-Gaussian model (eq. 19 in the main text). Experimental and predicted data matched perfectly (Fig. S7, panels b and c). Figure S6, panel d, and Figure S7, panel d, reflect the typical shape of a sigmoid as was not necessarily expected, because each point of the curve refers to a different R_0 value and, at a constant r_L, to a different ratio R_0/r_L and, by that, to different X_0- and M-values. Obviously, the relatively small R_0 variation within the analyzed data set (9.6 - 10.4 Å) left the overall picture of a sigmoid intact. DI-values ranged from about 10% to 80% (8.6% to 77.9%). This large diffusion-influence range that was experimentally covered speaks to the validity of the global analysis.

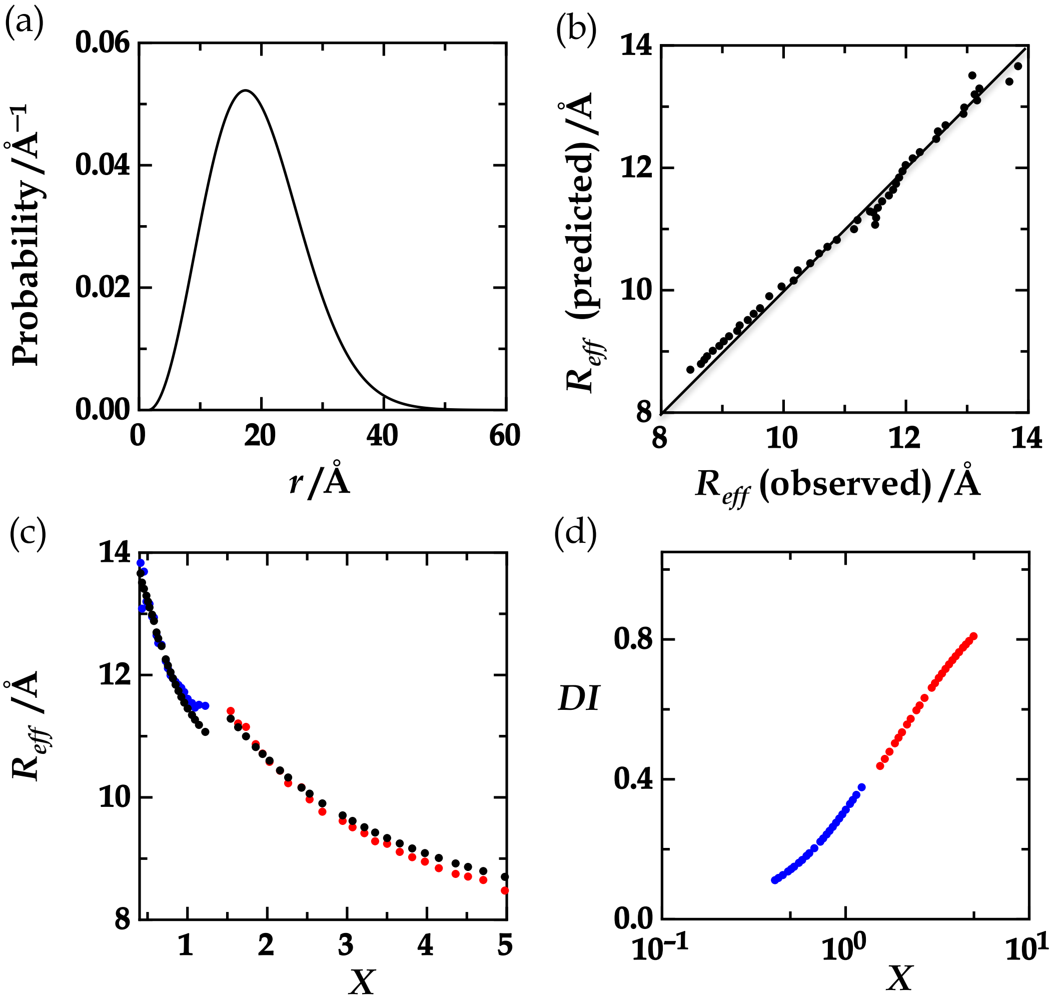

Figure S6. Results of a HSJ-based global fit of 48 experimental data sets. The "ideal chain" distance distribution model was used (eq. 18 in the main text) **(a)** The probability density distance distribution obtained from the ideal-chain analysis (solid line). The obtained parameters were b=19.5Å, D=68.5Å^2/ns, and r_L=1.5Å.

(b) The predicted effective distance plotted against the experimentally observed effective distance. The standard deviation from a straight line of slope one equaled 0.15Å. **(c)** The observed effective distance obtained from FTrp-sdFRET measurements (blue circles) and NAla-sdFRET measurements (red circles) and the predicted effective distance (black circles) plotted against X, the square root of the augmented diffusion coefficient, J. **(d)** The diffusion-influence values, obtained after normalization, plotted against X.

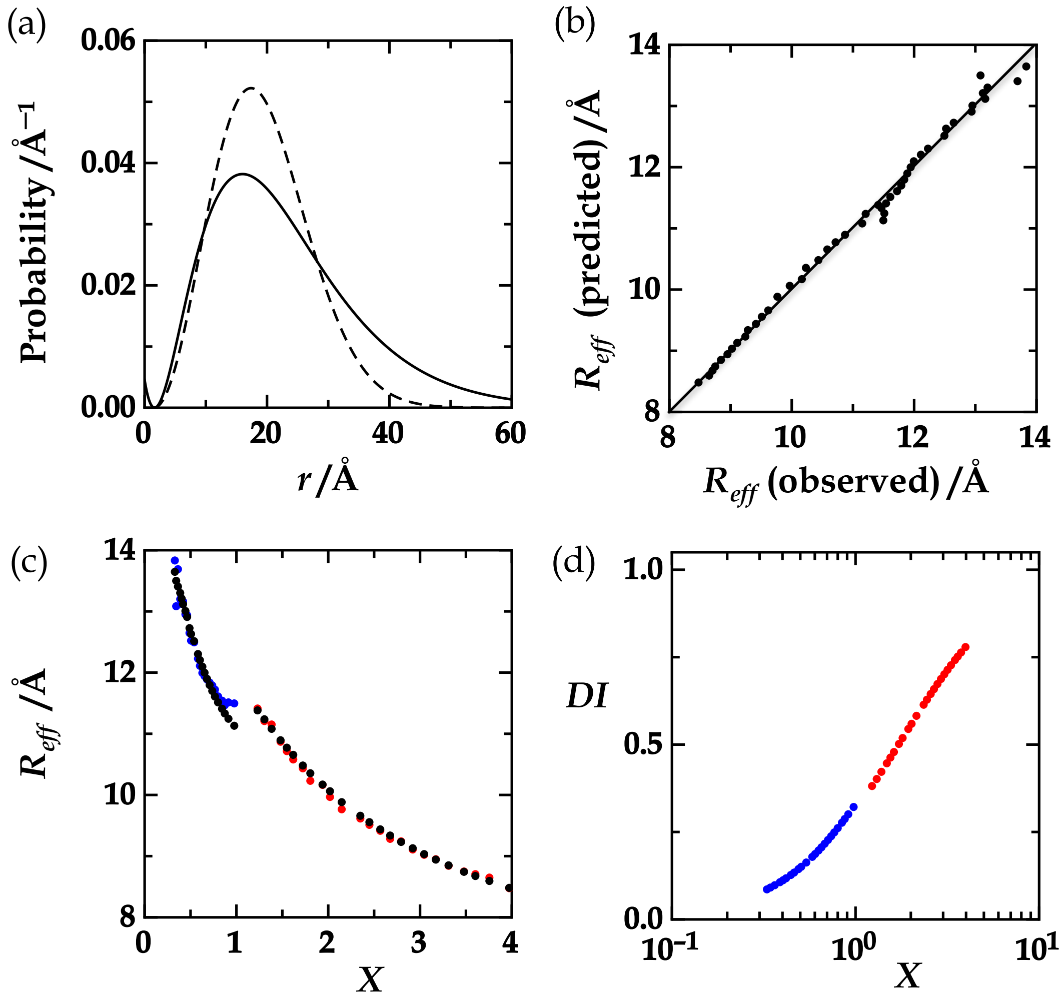

Figure S7. Results of a HSJE-based global fit of 48 experimental data sets. The 3D- or skewed-Gaussian model (eq. 19 in the main text) was used. The global fit resulted in a=9.6·10^{-6}Å^{-2}, b=−7221.85Å, D=43.4Å^2/ns and r_L=1.6Å. **(a)** The distance distribution obtained from the skewed-Gaussian fit (solid line) compared to the distribution obtained from the ideal-chain fit (dashed line). **(b)** The values of the predicted effective distance were plotted against the values of the experimentally observed effective distance. The standard deviation from a straight line of slope one equaled 0.12Å. **(c)** The observed effective distance obtained from FTrp-sdFRET measurements (blue circles) and NAla-sdFRET measurements (red circles) and the predicted effective distance (black circles) plotted against X, the square root of the augmented diffusion coefficient, J. **(d)** The diffusion-influence values, obtained after normalization, plotted against X.

We note the somewhat different diffusion coefficients obtained with the ideal-chain model (D=68.5Å^2/ns) and the skewed-Gaussian model (D=43.4Å^2/ns). In comparison to the ideal-chain distribution (Fig.S7a, solid line line) with its model-enforced symmetry around its peak,

the skewed-Gaussian model leads to a better, nearly perfect fit (Fig, S7b,c), and the distribution is slightly skewed to the left (Fig.S7a, dashed line). We understand the different diffusion constants when we recall that shorter donor-acceptor distances contribute more than longer distances to FRET (Förster's law, equation 1 in the main text), and that mutual donor-acceptor diffusion contributes more to DFRET when it is active at shorter distances. Thus, the Gaussian distribution curved towards shorter distances in combination with the smaller coefficient 43.4 Å^2/ns corresponds to the ideal-chain distribution, which peaks at a larger distance, in combination with the higher coefficient of 68.5 Å^2/ns. In an earlier work, we applied the HSE in a global analysis of the same data set and settled on a diffusion value of 53.4 ± 6.0 Å.[3] At that time, we were still unaware of the importance of the left integration limit, which we simply set to 0 Å. We see here very well that the "true" value of D depends on a correctly chosen distribution model. Thus, although we believe we are closer to the truth with respect to the distance distribution and diffusion coefficient, we also believe that further investigation into this matter is warranted. Indeed, it is also possible that a combination of two Gaussian distributions, resulting in a distribution with two peaks or one with a left shoulder, might better describe the experimental situation and also allow a somewhat higher, physically more intuitive value of the left-integration limit.

The HSJE allows for such a more complex analysis, leading to the final question of how to accurately determine an appropriate set of measurements that provides confidence in $p(r)$ and D. The structure of the augmented diffusion coefficient, J, clearly points to what should be part of the best strategy, the variation of R_0 (eq. 12 in the main text, eq. S33). Ideally, one would employ two different FRET donor-acceptor pairs in combination with donor lifetime and viscosity variation. It might even be possible to include a slight viscogen-induced change in the distance distribution into such an extended analysis.

9. Donor Quantum Yield

Indeed, there is a further photophysical parameter that can be included into the discussion, the donor-quantum yield. This parameter defined by equation S31 is implicit in the survey of FRET processes in Figure 1 in the main text. It is also among the topics in a couple of earlier DFRET studies [1,3,7,9]. The donor lifetime depends on the donor quantum yield and is the

product of the donor quantum yield and the radiative donor lifetime, τ_{rad}, the lifetime when the quantum yields equals unity (eqs. S31). The Förster radius also depends on the donor-quantum yield: The sixth power of the Förster radius is the product of the quantum yield and the sixth power of the critical Förster radius, the Förster radius when the quantum yield equals unity (eq. S32). Using equations S31 and S32, we obtain J and its square root X as function of the donor-quantum yield (S33).

$$\Phi_{\mathrm{D}} = \frac{k_{\mathrm{rad}}}{k_{\mathrm{rad}} + k_{\mathrm{nrad}}} = \frac{k_{\mathrm{rad}}}{k_{\mathrm{D}}} = \frac{\tau_{\mathrm{D}}}{\tau_{\mathrm{rad}}} \rightarrow \tau_{\mathrm{D}} = \Phi_{\mathrm{D}} \cdot \tau_{\mathrm{rad}} \tag{S31}$$

$$R_0^6 = \Phi_{\mathrm{D}} \cdot R_{\mathrm{F}}^6 \rightarrow R_0 = \Phi_{\mathrm{D}}^{1/6} \cdot R_{\mathrm{F}} \tag{S32}$$

$$J = \frac{D\tau_{\mathrm{D}}}{R_0^2} = \frac{\Phi_{\mathrm{D}} D\tau_{\mathrm{rad}}}{\left(\Phi_{\mathrm{D}}^{1/6} \cdot R_{\mathrm{F}}\right)^2} = \Phi_{\mathrm{D}}^{2/3} \frac{D\tau_{\mathrm{rad}}}{R_{\mathrm{F}}^2} \rightarrow X = \Phi_{\mathrm{D}}^{1/3} \frac{\sqrt{D\tau_{\mathrm{rad}}}}{R_{\mathrm{F}}} \tag{S33}$$

The donor-quantum yield seems to be an interesting variable as it can be independently controlled in experiments. Previously, we varied it by a factor of 12.6 by simply adding iodine ions to the the experimental samples [1]. However, we detected not the slightest effect on the effective distance. Due to the present study, we now know that we worked, at that time, close to the dynamic limit. A look at Figures S6d or S7d could suggest that decreasing the quantum yield and, by that, the augmented-diffusion coefficient, should decrease the diffusion influence and, by that, the effective distance. The experimental results, however, contradict such a gut feeling [1]. We now have learned that a decreasing quantum yield implies a decreasing Förster radius and, by that, an increasing M-value or slope of the DI-sigmoid; higher DI-values are reached earlier: When we are close to the dynamic limit, a quantum-yield variation has simply no impact on the diffusion influence and the effective distance. We need to remind ourselves that the dynamic limit is given by $R_{\mathrm{eff}}=R$ and that R does not depend on the donor quantum yield (eq. S20). In contrast, the static limit, $R_{\mathrm{eff}}=L$, depends on R_0 and, by that, on the quantum yield (eq. S19, eq. S32). Thus, a variation of the donor-quantum yield near to the static limit might feed additional information into a global analysis. However, we need to consider that the only signal measured in experiment is the fluorescence signal. When we significantly decrease the donor quantum yield, we also accept

a deteriorating fluorescence signal: The envisaged gain of information is offset by the loss in signal accuracy. Theoretically, quantum-yield variation could be useful for a global analysis, practically, it almost never is. In contrast, in another method based on two fluorescence labels, on two pyrene labels used in the framework of the FBM, of the fluorescence blob model, quantum-yield variation often played an important role [10-13].

We underestimated the role of the donor quantum yield when we previously proclaimed an equal or symmetrical influence of the diffusion coefficient and the *radiative* donor lifetime, τ_{rad}, on the measured effective distance [3]. When we put trust into the HSE, equivalence statement 2 (ES2) follows by force: the symmetry between the diffusion coefficient and the *experimental* donor lifetime, τ_D. In our previous analysis [3], quantum-yield variation and the distinction between radiative and experimental donor lifetime indeed made little difference in regard to the conclusions but we can not yet exclude that experimental circumstances might exist or can be created where it does.

10. Mathematical Challenges

a. We put much more effort in collecting evidence for the validity of ES1 than for the validity of ES2. The reason is that ES2 is a mathematically-enforced consequence of the HSE. To show that ES1 follows by force from the HSE still poses a challenge.

b. We observed perfectly symmetrical diffusion profiles ($DI(J)$, $DI(X)$) in our study but we were not yet able to come up with a proof that this has to be the case.

c. We were surprised that it is possible to derive a closed analytical expression from a PDE in a way that to the professional mathematician might appear as "child's play". We are therefore curious whether such a procedure could be extended to other PDEs to help the biologist/chemist/physicist to tackle her or his problem in a more efficient manner.

11. Outlook

The work ahead includes the extension of the HSJE to other ranges of R_0, that is, to other FRET donor-acceptor pairs. Further, guided by the structure of the augmented diffusion coefficient, we can now develop recipes to minimize and optimize experimental efforts to

determine polymer structure and dynamics, distance distribution and diffusion coefficient, by applying the HSJE in global analyses. It is also much easier now to determine experimental data sets that allow for the analysis of more complicated distance distributions such as a double-Gaussian distribution or a distribution that changes with the concentration of an added agent.

12. References

1. Jacob, M. H.; Dsouza, R. N.; Ghosh, I.; Norouzy, A.; Schwarzlose, T.; Nau, W. M. Diffusion-Enhanced Forster Resonance Energy Transfer and the Effects of External Quenchers and the Donor Quantum Yield *J. Phys. Chem. B* **2013**, *117*, 185-198.
2. Jacob, M. H.; Nau, W. M. In *Folding, Misfolding and Nonfolding of Peptides and Small Proteins*; Schweitzer-Stenner, R., Ed.; John Wiley & Sons: New Jersey, 2012.
3. Jacob, M. H.; Ghosh, I.; D'Souza, R. N.; Nau, W. M. Two Orders of Magnitude Variation of Diffusion-Enhanced Förster Resonance Energy Transfer in Polypeptide Chains *Polymers* **2018**, *10*, 1-15.
4. Jacob, M. H.; D'Souza, R. N.; Schwarzlose, T.; Wang, X.; Huang, F.; Haas, E.; Nau, W. M. Method-Unifying View of Loop-Formation Kinetics in Peptide and Protein Folding *J. Phys. Chem. B* **2018**, *122*, 4445-4456.
5. Maliwal, B. P.; Kusba, J.; Wiczk, W.; Johnson, M. L.; Lakowicz, J. R. End-to-End Diffusion Coefficients and Distance Distributions from Fluorescence Energy Transfer Measurements: Enhanced Resolution by Using Multiple Acceptors with Different Förster Distances *Biophys. Chem.* **1993**, *46*, 273-281.
6. Haas, E. The Study of Protein Folding and Dynamics by Determination of Intramolecular Distance Distributions and Their Fluctuations Using Ensemble and Single-Molecule Fret Measurements *ChemPhysChem* **2005**, *6*, 858-870.
7. Toptygin, D. Effect of Diffusion on Resonance Energy Transfer Rate Distributions: Implications for Distance Measurements *J. Phys. Chem. B* **2015**, *119*, 12603-12622.
8. This weighing procedure is routinely used whenever, f. i., Tryptophan serves as donor. Tryptophan usually leads to multiexponential time courses as the lifetimes of its rotamer states differ However, also the donor quantum yield of these states differ, which is why ref. S7 exposes this weighing procedure as a rough approximation.
9. Lakowicz, J. R.; Kusba, J.; Gryczynski, I.; Wiczk, W.; Szmacinski, H.; Johnson, M. L. End-to-End Diffusion and Distance Distributions of Flexible Donor-Acceptor Systems Observed by Intramolecular Energy-Transfer and Frequency-Domain Fluorometry - Enhanced Resolution by Global Analysis of Externally Quenched and Nonquenched Samples *J.Phys. Chem.* **1991**, *95*, 9654-9660.
10. Duhamel, J. Polymer Chain Dynamics in Solution Probed with a Fluorescence Blob Model *Acc. Chem. Res.* **2006**, *39*, 953-960.
11. Ingratta, M.; Duhamel, J. Effect of Time on the Rate of Long Range Polymer Segmental Intramolecular Encounters *J. Phys. Chem. B* **2009**, *113*, 2284-2292.
12. Chen, S.; Duhamel, J.; Winnik, M. A. Probing End-to-End Cyclization Beyond Willemski and Fixman *J. Phys. Chem. B* **2011**, *115*, 3289-3302.
13. Duhamel, J. Global Analysis of Fluorescence Decays to Probe the Internal Dynamics of Fluorescently Labeled Macromolecules *Langmuir* **2014**, *30*, 2307-2324.
